# A quantum coherent spin in hexagonal boron nitride at ambient conditions

**DOI:** 10.1038/s41563-024-01887-z

**Published:** 2024-05-20

**Authors:** Hannah L. Stern, Carmem M. Gilardoni, Qiushi Gu, Simone Eizagirre Barker, Oliver F. J. Powell, Xiaoxi Deng, Stephanie A. Fraser, Louis Follet, Chi Li, Andrew J. Ramsay, Hark Hoe Tan, Igor Aharonovich, Mete Atatüre

**Affiliations:** 1 Cavendish Laboratory, University of Cambridge, Cambridge, UK; 2 Photon Science Institute and Department of Physics and Department of Chemistry, The University of Manchester, Manchester, UK; 3 Hitachi Cambridge Laboratory, Hitachi Europe Ltd, Cambridge, UK; 4 School of Mathematical and Physical Sciences, Faculty of Science, University of Technology Sydney, Ultimo, New South Wales Australia; 5 ARC Centre of Excellence for Transformative Meta-Optical Systems, Faculty of Science, University of Technology Sydney, Ultimo, New South Wales Australia; 6 ARC Centre of Excellence for Transformative Meta-Optical Systems, Department of Electronic Materials Engineering, Research School of Physics, The Australian National University, Canberra, Australian Capital Territory Australia

**Keywords:** Two-dimensional materials, Qubits, Single photons and quantum effects, Quantum optics

## Abstract

Solid-state spin–photon interfaces that combine single-photon generation and long-lived spin coherence with scalable device integration—ideally under ambient conditions—hold great promise for the implementation of quantum networks and sensors. Despite rapid progress reported across several candidate systems, those possessing quantum coherent single spins at room temperature remain extremely rare. Here we report quantum coherent control under ambient conditions of a single-photon-emitting defect spin in a layered van der Waals material, namely, hexagonal boron nitride. We identify that the carbon-related defect has a spin-triplet electronic ground-state manifold. We demonstrate that the spin coherence is predominantly governed by coupling to only a few proximal nuclei and is prolonged by decoupling protocols. Our results serve to introduce a new platform to realize a room-temperature spin qubit coupled to a multiqubit quantum register or quantum sensor with nanoscale sample proximity.

## Abstract (web summary)

Quantum coherent control of single-photon-emitting defect spins have been reported in hexagonal boron nitride, revealing that spin coherence is mainly governed by coupling to a few proximal nuclei and can be prolonged by decoupling protocols.

## Main

Scalable spin–photon quantum interfaces require single-photon generation with a level structure that enables optical access to the electronic spins^1–3^. These systems are critical for the deployment of applications such as quantum repeaters^4–6^ and quantum sensors^7–10^. Ideally, a spin–photon interface should display long-lived spin coherence with efficient and coherent optical transitions in a scalable material platform without requiring stringent operation conditions such as cryogenic temperature or applied magnetic field. Materials that host atomic defects with well-defined optical and spin transitions garner the most attention. Several systems have been studied in detail^11–15^, but only a few individually addressable defects in diamond and silicon carbide possess quantum coherent spins at room temperature, although with challenging optical properties^2,16,17^. Realizing the ideal spin–photon interface requires engineering existing candidates for better performance, as well as exploring new material systems^3,18^.

Layered materials have emerged as a new quantum platform where their natural suitability for large-area growth, deterministic defect creation and hybrid device integration may enable accelerated scalability^19–23^. Hexagonal boron nitride (hBN) is a wide-bandgap (~6 eV) layered material that hosts a plethora of lattice defects that emit across the visible and near-infrared spectral regions. The first spin signature for hBN defects came from ensembles attributed to a boron vacancy (V_B_^−^) with a broad optical emission spectrum centred at ~800 nm (refs. ^24,25^). These defects have been subject to spin initialization and manipulation, although exclusively on an ensemble level due to their low optical quantum efficiency^26–29^. In contrast, defects in the visible spectrum (~600 nm) generate bright, tuneable, single-photon emission of up to 80% into the zero-phonon line at room temperature^30–34^. Preliminary reports on these optically isolated defects captured spin signatures via optically detected magnetic resonance (ODMR), but these signatures are only present under a finite magnetic field, indicating either a spin-triplet (*S* = 1) with low zero-field splitting or a spin-half (*S* = 1/2) system^35–37^.

In this work, we implement room-temperature coherent spin control of individually addressable single-photon-emitting defects in hBN. We reveal a spin-triplet (*S* = 1) ground-state spin manifold with 1.96 GHz zero-field splitting using angle-resolved magneto-optical measurements. We observe that the principal symmetry axis of the defect lies in the plane of the hBN layers, indicating a low-symmetry chemical structure. Microwave-based Ramsey interferometry reveals an inhomogeneous dephasing time ${T}_{2}^{\,* }$ of ~100 ns. Interestingly, the continuously driven spin Rabi coherence time (*T*_Rabi_) is prolonged beyond 1 μs at room temperature with no magnetic field. This drive-prolonged coherence time indicates that the electronic spin can be protected from its reversibly decohering environment, the nuclei. We confirm this via standard dynamical decoupling pulse protocols yielding a spin-echo coherence time (*T*_SE_) of ~200 ns, which exceeds ~1 μs with ten refocusing pulses. The scaling of the coherence time with the number of decoupling pulses, together with the fine structure of the ODMR signal, suggests hyperfine coupling to only a few inequivalent nitrogen and boron nuclei.

## A ground-state electronic spin triplet

**Fig. 1:**
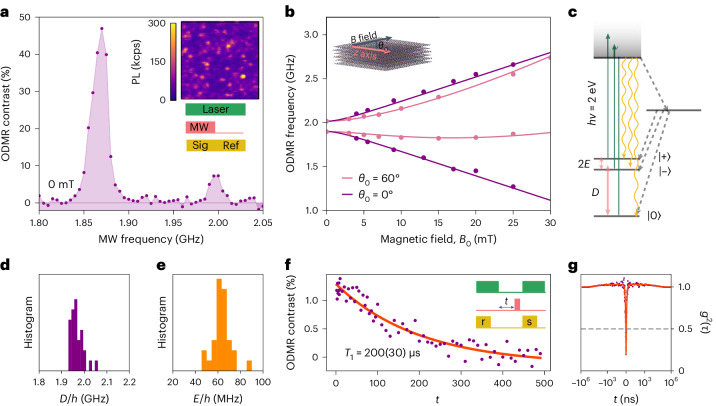
A ground-state spin triplet. **a**, An ODMR spectrum of a single defect in hBN measured in the absence of a magnetic field. The top-right inset shows a confocal image of the PL intensity of the hBN device under 532 nm laser illumination. Scale bar, 0–300 kcps. The bottom-right inset shows the measurement sequence. **b**, ODMR resonance frequencies for a defect, where the magnetic field is applied in the plane of the hBN layers along the defect *z* axis (*θ*_0_ = 0°) (purple circles) and 60° from the *z* axis (*θ*_0_ = 60°) (pink circles). Both measurements are fit with an *S* = 1 model using equation (1) (purple and pink curves, respectively). The inset defines the orientation of the magnetic field relative to the defect *z* axis and the hBN layers, identified by *θ*_0_. **c**, Inferred level structure for the defect, displaying a spin-triplet ground state and a spin-singlet metastable state. The green arrows show optical excitation; yellow arrows, PL; black arrows, intersystem crossing; and pink arrows, microwave (MW) drive. **d**,**e**, Statistical distribution of the *D* and *E* zero-field splitting parameters obtained from 25 defects. **f**, Spin–lattice relaxation time (*T*_1_) measurement of the ground-state spin, measured in the absence of a magnetic field. The inset shows the microwave measurement sequence (not drawn to scale), where r is the reference readout, s is the signal readout and t is the pulse delay. **g**, Second-order intensity-correlation measurement (*g*^(2)^(t)) of a single defect.

We investigate multilayer hBN that is grown via metal–organic vapour phase epitaxy using a carbon precursor and ammonia^38^, resulting in single-photon-emitting and spin-active defects that are related to carbon^38,39^. The flow rate of the carbon precursor determines the defect density in hBN and a 10 μmol min^−1^ flow rate yields an individually addressable defect density of ~1 defect per μm^2^. Figure 1a (top-right inset) shows a confocal scan image of the photoluminescence (PL) of the hBN material under 532 nm laser illumination (Supplementary Figs. 1 and 2 and Supplementary Section 1.3). The same material was the subject of a previous investigation that identified emitters displaying single-spin resonance under a magnetic field, assigned to an *S* > 1/2 system with low zero-field splitting^36^. To identify the ground-state spin resonances, we measure continuous-wave ODMR, using the protocol presented in Fig. 1a (bottom-right inset). Figure 1a shows an example ODMR spectrum for a single defect in the absence of the applied magnetic field and at room temperature. The ODMR signal displays strikingly strong contrast reaching nearly 50%. Unlike all the previous reports on single-spin-active hBN defects, the spectrum shows two distinct resonances, namely, *υ*_1_ = 1.87 GHz and *υ*_2_ = 1.99 GHz, in the absence of a magnetic field. We note that some defects also show the previously reported^36^ ODMR resonance with low zero-field splitting when a magnetic field is applied (Supplementary Figs. 8–10). We confirm that the presence of both resonances cannot be explained by a single-spin model (Supplementary Section 4), indicating the possibility that these two resonances relate to different charge or electronic states of the same defect.

The observation of a zero-field resonance unambiguously determines a spin multiplicity of >½, indicating that this spin resonance is distinct from previous reports^35,37^. We assign the two resonances to the expected transitions of a spin triplet with lifted three-fold degeneracy between the spin sublevels, ruling out higher spin configurations (Supplementary Section 4). An effective-spin Hamiltonian describes the eigenstates of the *S* = 1 system, which, in the absence of hyperfine coupling, takes the following form:

1 $$H=D({S}_{\rm{z}}^{2}-S(S+1)/3)+E({S}_{\rm{x}}^{2}-{S}_{\rm{y}}^{2})+({\,g}_{{\rm{e}}}{\mu }_{{\rm{B}}}){\bf{B}}{\bf{\bullet }}{\bf{S}},$$

where **S** is the spin vector with projection operators *S*_*x*_, *S*_*y*_ and *S*_*z*_; *g*_e_ is the free electron *g*-factor, *μ*_B_ is the Bohr magneton and **B** is the magnetic field with magnitude *B*_0_. The zero-field transitions are characterized by the parameters *D* and *E*, where *υ*_1,2_ = (*D* ± *E*)/*h* and *h* is Planck’s constant. The ODMR spectrum (Fig. 1a) yields *D*/*h* = 1.930(10) GHz and *E*/*h* = 60(10) MHz. To confirm the *S* = 1 assignment, we perform vector-magnetic-field-dependent ODMR measurements. Figure 1b displays an example of the dependence of the ODMR transition frequencies as the external magnetic field is applied at *θ*_0_ = 0° (dark purple circles) and *θ*_0_ = 60° (light pink circles), where *θ*_0_ is the angle between **B** and the defect *z* axis. We determined that the defect *z* axis lies in the plane of the hBN layers (Fig. 1b, inset), for all the defects that we study, with a confidence of ~18°, via a series of angular ODMR measurements (Supplementary Figs. 11–14). The purple and pink curves show the simulated transition frequencies between the eigenstates of the *S* = 1 spin Hamiltonian of equation (1), with *D*/*h* = 1.959 GHz and *E*/*h* = 59 MHz. The same model effectively captures the appearance of the anticipated |+〉 to |−〉 (where |±〉 = 2^−1/2^|(+1 ± −1)〉) transition in the high off-axis-field regime. Interestingly, the ODMR contrast is not quenched up to >100 mT magnetic field applied orthogonal to the defect *z* axis—the highest magnetic-field strengths we access (Supplementary Fig. 16). Figure 1c illustrates the assigned energy-level structure, complete with a ground-state *S* = 1 system with 1.96 GHz zero-field splitting and a metastable spin-singlet state responsible for off-resonance optical spin initialization^16^. We measure the ground-state resonance across more than 25 defects across multiple devices and obtain a narrow distribution of *D* and *E* values: *D*/*h* = 1.971(25) GHz and *E*/*h* = 62(10) MHz (Fig. 1d,e). Due to sufficiently large zero-field splitting parameters, each electronic spin resonance can be independently addressed. Therefore, Fig. 1f presents an example measurement of the spin–lattice relaxation (*T*_1_) time, where the inset shows the protocol used for pulsed ODMR, including a calibrated sequence of laser, microwave and readout pulses for spin initialization, spin control and spin readout, respectively (Supplementary Figs. 18 and 19). The measured *T*_1_ values vary in the range of 35–200 μs, which is long compared with the optically excited-state lifetime of ~5 ns (Supplementary Fig. 4) and that of the previously identified resonance (~9 μs (Supplementary Fig. 20)). We conclude that this resonance arises from a ground-state *S* = 1 system. Figure 1g shows an example second-order intensity-correlation measurement, *g*^(2)^(t), of this defect (Supplementary Figs. 3 and 4 show additional defects).

## Spin coherence and protection under ambient conditions

**Fig. 2:**
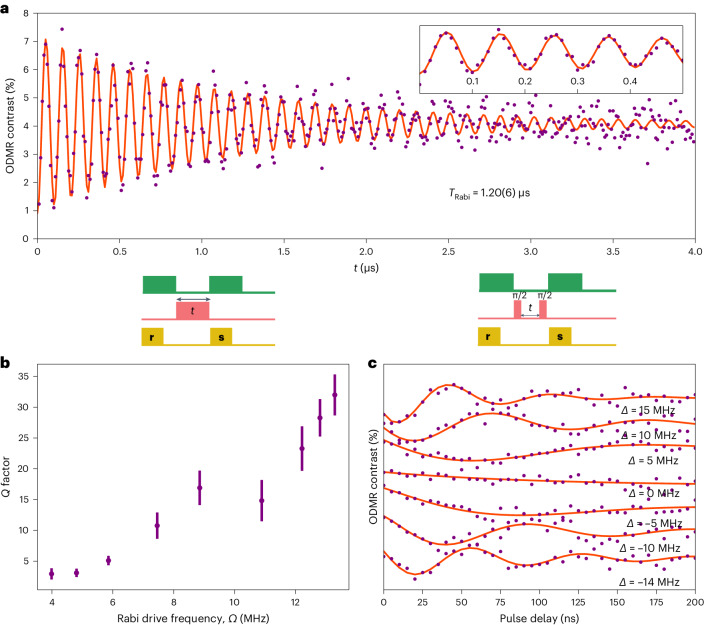
Spin coherence and protection. **a**, Rabi oscillations (purple circles) for a single hBN defect measured at high microwave power, fit to a function of the form exp(−*t*/*T*_Rabi_)sin(2π*tΩ* − *φ*) (orange curve), where *φ* is the phase offset, *Ω* is the Rabi frequency and *T*_Rabi_ is the decay lifetime of the Rabi oscillations. The inset shows a zoomed-in view of the data. **b**, *Q* factor of the Rabi oscillations as a function of the Rabi frequency, where *Q* = *T*_Rabi_/*T*_π_ and *T*_π_ = 1/2*Ω*. The presented data (purple circles) are calculated using *T*_Rabi_ and *T*_π_ extracted from fits to Rabi measurements using the equation above. The error bars are obtained from the 68% confidence intervals of each fit parameter. The Rabi measurement sequence is drawn above the panel (not drawn to scale), where **r** is the reference readout, **s** is the signal readout and *t* is the pulse duration. **c**, Ramsey measurements for different values of microwave frequency detuning *Δ*, from the resonance. The red curves are fit to exp(–${t}{/}{{T}}_{{2}}^{{\,* }}{}$)sin(2π*τΩ*_Ramsey_ − *φ*). The diagram above the panel shows the microwave measurement sequence (not drawn to scale), where **r** is the reference readout, **s** is the signal readout and *t* is the pulse delay. All the measurements are measured in the absence of a magnetic field.

Figure 2a shows example spin Rabi oscillations, where we vary the duration of a resonant microwave pulse and measure the ODMR contrast. The data (purple circles) are fit to a function of the form exp(−*τ*/*T*_Rabi_)sin(2π*τΩ* − *φ*) (red curve), where *φ* is the phase offset, *Ω* is the Rabi drive frequency and *T*_Rabi_ is the lifetime of Rabi oscillations. We confirm that *Ω* shows a linear dependence on the square root of the microwave power (Supplementary Fig. 21), as expected. Surprisingly, the oscillations persist for over a microsecond (*T*_Rabi_ = 1.20(6) μs), yielding a π-pulse fidelity of 0.96(2) and a quality factor (*Q* = *T*_Rabi_/*T*_π_) of 24(3). However, as highlighted by the example shown in Fig. 2b, *T*_Rabi_ also displays a notable prolongation as a function of *Ω*—a signature behaviour for a spin-rich environment, observed in other III–V materials^40,41^. In this regime, the strongly driven spin is effectively decoupled from a slowly evolving nuclear environment akin to motional narrowing^42^.

To obtain the bare inhomogeneous dephasing time (${{T}}_{{2}}^{{\,* }}$) for the single defect, we perform microwave-based Ramsey interferometry. Figure 2c presents the Ramsey measurements for five values of detuning *Δ* between the microwave drive frequency and *υ*_1_ transition. Here the red curves are fit to the data using exp(–${t }{/}{{T}}_{{2}}^{{\,* }}{}$)sin(2π*τΩ*_Ramsey_ − *φ*), where *Ω*_Ramsey_ is the frequency of oscillations arising from *Δ*. We extract a collective value for ${{T}}_{{2}}^{{\,* }}$ of 106(12) ns, commensurate with the ~10 MHz linewidth of the unsaturated ODMR spectrum.

**Fig. 3:**
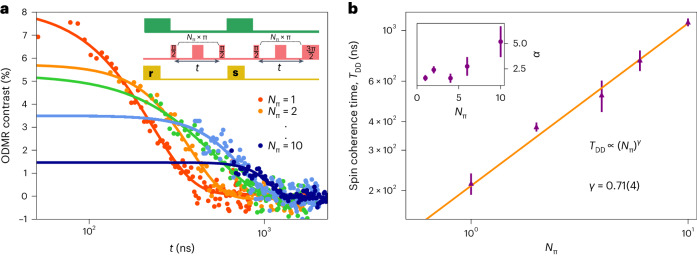
Scaling of spin coherence under dynamical decoupling. **a**, Dynamic decoupling measurements with *N*_π_ refocusing pulses, where each measurement is fit to exp[−(*t*/*T*_DD_)^*α*^]. The measurement sequence is shown in the inset, where **r** is the reference readout, **s** is the signal readout and *t* is the pulse delay. **b**, Spin coherence time *T*_DD_ (purple triangles) as a function of the number of refocusing pulses *N*_π_. The orange curve is fit to a power law, $\sim {{N}_{\uppi }}^{\gamma }$. The data (purple triangles) are extracted from fits to the data shown here. The error bars indicate the 68% confidence interval. All the measurements are performed in the absence of applied magnetic field.

Figure 3a presents spin coherence measurements using a basic multipulse dynamical decoupling protocol, without phase control or pulse corrections (measurement sequence illustrated in the inset). Here spin-echo measurements (red circles), comprising a single refocusing pulse, *N*_π_ = 1, yields *T*_SE_ = 228(11) ns and *α* = 1.81(22) by fitting to exp[−(*τ*/*T*_SE_)^α^] (red curve). This value is comparable with *T*_SE_ ≈ 100 ns determined for V_B_^−^-defect ensembles in hBN under equivalent conditions^26,29^. We further prolong the spin coherence time by including additional refocusing pulses. Figure 3a displays a range of measurements (*N*_π_ = 2, 4, 6, 10) plotted in orange-to-blue colour-coded data (circles) and the corresponding fits (curves). The drop in ODMR contrast with increasing *N*_π_ is due to the limited π-pulse fidelity in the multipulse experiment. Figure 3b presents the dependence of the protected spin coherence time *T*_DD_ as a function of *N*_π_, reaching 1.08(4) µs with ten refocusing pulses. The inset shows the evolution of *α* values extracted from Fig. 3a fits as a function of *N*_π_, staying above 2 for all the values and reaching ~5 for 10 pulses, indicating strong protection. The orange curve (Fig. 3b) is a power-law fit yielding a *T*_DD_ scaling of ${{N}}_{{\uppi }}^{{0}{.}{71}{(}{4}{)}}$. This scaling exponent falls close to the 0.67 scaling signature expected theoretically for a central electronic spin interacting weakly with a few slowly evolving proximal nuclei^43,44^.

## Hyperfine signatures of proximal nuclei

**Fig. 4:**
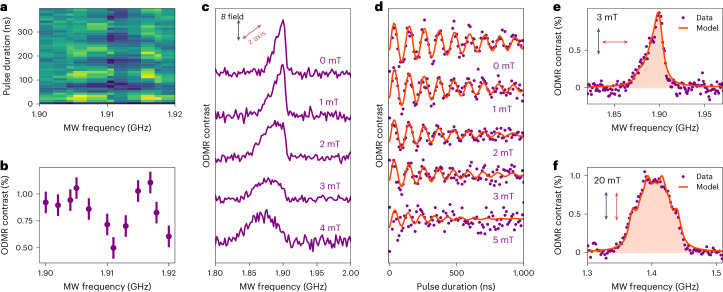
Hyperfine signatures in the ODMR spectrum. **a**, Rabi oscillations as a function of microwave frequency, measured at low microwave power. **b**, Linecut of the data in **a**, integrated over the first 100 ns. The presented data are the mean ± one standard deviation; *N* = 10. **c**,**d**, ODMR spectra and Rabi oscillations measured between 0 and 5 mT oriented at an arbitrary angle to the defect *z* axis. **e**, ODMR spectrum measured for 3 mT magnetic field orthogonal to the defect *z* axis. **f**, ODMR spectrum measured with 20-mT magnetic field parallel to the defect *z* axis. The shaded curves in **e** and **f** are the computed spectra (Supplementary Section 4).

To understand the nature of hyperfine coupling and gain an insight into the chemical structure of the defect, we investigate the *υ*_1_ resonance for the presence of spectral fine structure. Figure 4a presents a map of Rabi oscillations as a function of microwave detuning, performed in the absence of applied magnetic field and below saturation, where a multipeak structure is evident. An integrated linecut of this map between 0 and 100 ns reveals two maxima separated by approximately 10 MHz (Fig. 4b). This value is commensurate with the unsaturated ODMR linewidth and the 1/${T}_{2}^{\,* }$ value. Figure 4c,d presents the unsaturated ODMR spectra and Rabi oscillations, respectively, performed with the magnetic field oriented ~60° with respect to the defect *z* axis. The unsaturated zero-field ODMR spectrum is narrow and asymmetric with a linewidth of the order of 10 MHz (Fig. 4 and Supplementary Fig. 22). With increasing magnetic field, the ODMR spectrum broadens, and we observe faster dephasing of Rabi oscillations. The purple circles in Fig. 4e show the ODMR spectrum for 3-mT magnetic field orthogonal to the *z* axis (*θ*_0_ = 90°). In this configuration, in contrast to T field applied at 60°, the ODMR spectrum remains narrow and asymmetric as that in the absence of a magnetic field. Figure 4f presents the ODMR spectrum for 20 mT magnetic field applied parallel to the *z* axis (*θ*_0_ = 0°). In this case, the ODMR lineshape is symmetric and structured, indicating a discrete number of overlapping hyperfine resonances (Supplementary Section 4). The orange-shaded curves under the ODMR spectra (Fig. 4e,f) are the ODMR spectra computed for an *S* = 1 electronic spin coupled to two inequivalent nuclear spins with hyperfine couplings of 13 and 22 MHz (Supplementary Information). These nuclear spins can be of different species (for example, one N and one B), or the same species with different hyperfine couplings (Supplementary Fig. 23). In fact, the computed spectrum (Fig. 4f) corresponds to hyperfine coupling to two ^14^N atoms. Strikingly, an *S* = 1 central spin coupled to two inequivalent nuclei is the only model that captures the magnetic-field amplitude and orientation dependence of the ODMR spectrum (Supplementary Fig. 24). These results, together with the in-plane symmetry of the defect, provide valuable insights for theoretical efforts aimed at determining the microscopic structure of this carbon-based spin-triplet defect^45–47^.

In the low-field regime, the fine structure of the ODMR spectrum is intricately linked to the low-symmetry character of the defect. For an *S* = 1 electronic spin, the non-zero transverse zero-field splitting *E* gives rise to an avoided crossing at zero magnetic field, which may be accompanied by the quenching of a relatively weaker hyperfine interaction. However, if the strength of the hyperfine coupling to neighbouring nuclei is comparable with *E*/*h*, then it introduces an asymmetric perturbation to the ODMR spectrum, as observed here. The presence of the avoided crossing effectively results in a clock transition with the corresponding protection of the electronic spin coherence at zero magnetic field. In the low-field regime, when the magnetic field is applied orthogonal to the defect *z* axis, the clock-transition character of the *υ*_1_ line is preserved, whereas a magnetic field applied parallel to the defect *z* axis brings the system away from the clock-transition point. Therefore, the ODMR spectrum shown in Fig. 4e is reminiscent of the zero-field spectrum of Fig. 4c, whereas the 3 mT spectrum shown in Fig. 4c is considerably broadened, compatible with our identification of a low-symmetry spin-triplet defect. We believe that the confirmation of a low-symmetry structure rules out single-site vacancies, single-atom substituents and carbon tetramers^47^ as potential chemical structures for this defect (Supplementary Section 4.3). Carbon trimers^45,48^ and vacancy-substituent complexes^49^ are low symmetry, but structures that have been theoretically considered to date are not predicted to display *S* = 1 with ~2 GHz zero-field splitting^48,49^. Larger carbon defects (namely, C_4_(*cis* and *trans*)) or complexes involving carbon or oxygen^50^ are potential candidates^51^, but these structures need to be investigated further.

Identification of a *D* = 1.96 GHz, *S* = 1 ground-state spin resonance for defects that simultaneously show a lower-frequency spin resonance, characterized previously on the same material^36^, indicates that these resonances are related. We conjecture that we identify the true ground state for a class of visible-emitting hBN defects, where previous reports of single-defect ODMR may have focused on the excited state, charge state or an alternative defect type. This is supported by our repeat identification of single defects that show both resonances with comparable contrast values and frequencies that cannot be explained by a single-spin model (Supplementary Sections 2.2 and 4). Alternatively, the identification of two spin resonances on a single defect could point towards complex charge state dynamics, which remains to be explored.

## Outlook

An optically addressable *S* = 1 defect with proximal nuclei displaying quantum coherence at room temperature and zero magnetic field in a layered material offers immense promise for quantum technologies. For quantum networks, this system can offer a feasible scaling of quantum repeater hardware under ambient operation conditions provided the necessary optical quality is delivered via integrated quantum photonics systems. The layered material nature of hBN may facilitate integration into nanostructures for tuning the spin and optical properties via the application of strain and electric fields without compromising quality. The proximal nuclear spins, coupled to the electronic spin, present an opportunity for the realization of long-lived quantum registers for the electronic spin qubit—a critical element for large-scale quantum architectures. For quantum sensing, this defect offers a unique and flexible system capable of nanoscale sensing under ambient conditions. The defects reported here are at most 15 nm away from any surface, highlighting their potential as highly proximal nanoscale sensors. We estimate a minimum detectable d.c. magnetic field of 3 μT Hz^–1/2^, using 0.77(*h*/*g*_e_*μ*_B_)${(}{\Delta }{\nu }{/}{C}\sqrt{{R}}{)}$ (ref. ^52^), where Δ*ν* is the linewidth (10 MHz), *C* is the contrast (30%) and *R* is the measure of brightness (10^5^ events per second). This estimate is of the same order as that of the well-established nitrogen-vacancy centres in diamond, with the added advantage of a system that is somewhat resilient to surface charge noise. Further, the retention of high ODMR contrast under an off-axis magnetic field offers flexibility on the dynamic range of vector magnetometry measurements.

## Methods

### Materials preparation

Multilayer hBN was grown by metal–organic vapour phase epitaxy on sapphire^36,38,39^. Briefly, triethyl boron and ammonia were used as boron and nitrogen sources, respectively, with hydrogen used as the carrier gas. Growth was performed at low pressure (85 mbar) and at a temperature of 1,350 °C, on sapphire substrates. Isolated defects were generated by modifying the flow rate of triethyl boron during growth, a parameter known to control the incorporation of carbon within the resulting hBN sheets^39^. The resulting material is ~30 nm thick. For confocal PL measurements, the hBN sheets were transferred to SiO_2_/Si substrates, using a water-assisted self-delamination process to avoid polymer contamination.

### Experimental setup

We perform optical measurements at room temperature under ambient conditions using a home-built confocal microscopy setup^36^. We use a continuous-wave 532 nm laser (Ventus 532, Laser Quantum) that is passed through a 532 nm band-pass filter, onto a scanning mirror and focused on the device using an objective lens with ×100 magnification and a numerical aperture of 0.9. We control the excitation power using an acousto-optic modulator (AA Optoelectronics), with the first-order diffracted beam fibre-coupled into the confocal setup. We remove any residual laser light from the collected emission using two 550 nm long-pass filters (Thorlabs FEL550). The remaining light was sent either into an avalanche photodiode (SPCM-AQRH-14-FC, Excelitas Technologies) for recording photon count traces or to a charge-coupled-device-coupled spectrometer (Acton Spectrograph, Princeton Instruments) via single-mode optical fibres (SM450 and SM600) for PL spectroscopy measurements. We carry out intensity-correlation measurements using a Hanbury Brown and Twiss interferometry setup using a 50:50 fibre beamsplitter (Thorlabs) and a time-to-digital converter (quTAU, qutools) with 81 ps resolution. We used a continuous-wave 405 nm laser (Thorlabs LP405-SF10) via a 405 nm dichroic mirror (Semrock BLP01-405R) to illuminate the sample for charge control.

### ODMR

We perform ODMR measurements using the confocal setup described above. A copper coil was placed in front of the hBN sample, with optical access through the coil, to provide an out-of-plane oscillating magnetic field. For continuous-wave ODMR measurements, a 70 Hz square-wave modulation was applied to the microwave amplitude to detect the change in PL counts as a function of microwave frequency. For pulsed ODMR, a calibrated pulse sequence or laser, microwave and readout pulses are used to determine the defect count rate with or without the presence of a spin-flipping microwave pulse. As for continuous-wave ODMR, the ODMR contrast (*C*) is given by *C* = *I*_sig_ – *I*_ref_/*I*_ref_, where *I*_sig_ and *I*_ref_ are the number of counts detected in the signal or reference, respectively. For measurements in the absence of an applied magnetic field, these were not measured in a shielded environment and therefore were susceptible to the Earth’s magnetic field and magnetic components on the optical table (~0.4 mT). For measurements under an applied magnetic field, we use a permanent magnet on a translation stage that can be changed in proximity and orientation relative to the device. We perform pulsed ODMR measurements using a pulse streamer (Swabian 8/2) to control a series of switches (Mini-Circuits ZYSWA-2-50DR+) to modulate the laser power, microwave power and signal readout duration.

## Online content

Any methods, additional references, Nature Portfolio reporting summaries, source data, extended data, supplementary information, acknowledgements, peer review information; details of author contributions and competing interests; and statements of data and code availability are available at 10.1038/s41563-024-01887-z.

## Supplementary information

Supplementary Information Supplementary Sections 1–4 and Figs. 1–24.

## Data Availability

All data needed to evaluate the conclusions in the paper are present in the Article or its Supplementary Information. All datasets are available from the corresponding authors upon reasonable request.

## Code availability

All codes used to generate the figures and analyse the data are available from the corresponding authors upon reasonable request.
