# Supplementary material for: A quantum coherent spin in hexagonal boron nitride at ambient conditions
Source: Nat Mater. 2024 May 20;23(10):1379–85. doi: 10.1038/s41563-024-01887-z (PMC11442369; doi:10.1038/s41563-024-01887-z)
Supplement: Supplementary file 1 — Supplementary Sections 1–4 and Figs. 1–24. [file 41563_2024_1887_MOESM1_ESM.pdf]

nature materials

Supplementry information https://doi.org/10.1038/s41563-024-01887-z

A quantum coherent spin in hexagonal boron nitride at ambient conditions

In the format provided by the authors and unedited

Contents

1.) Material characterization

1.1 Confocal setup and defect identification…………. ……………………………………………..…2
1.2 Second-order intensity-correlation ($g^{(2)}(t)$) measurements ………..……………………………...3
1.3 Integrated photoluminescence spectra……………………………………………………………...5

2.) ODMR measurements

2.1 Determination of microwave and optical saturation condition…………………………………..…7
2.2 Observation of the previously reported ODMR resonance ………………………………………...7
2.3 Magnetic field dependent ODMR…………………………………………………………………9
2.4 Zero-field ODMR………………………………………………………………………………….13

3.) Coherent microwave control

3.1 Readout calibration……….………………………………………………………………………14
3.2 T_1 measurements………………………………………………………………………………...14
3.3 Rabi measurements…………………………………………………………………………………15
3.4 Pulsed ODMR lineshape…………………………………………………………………….......16

4.) Theoretical modelling

4.1 Electron spin Hamiltonian model and *S*=3/2………………………………….………………..16
4.2 Hyperfine models…….……...16
4.3 Comments on potential chemical structures……….………………………………………….....18

5.) Appendix……………………………………………………………………………..…………..20

6.) References…………………………………………………….……………………………….…21

1. Material characterisation

1.1 Confocal setup and defect identification

We use the Qudi software *(1)* to identify ODMR-active defects via automated confocal scans with 100 μW 532-nm excitation, followed by ODMR measurements performed in the absence of an applied magnetic field. The process of identifying defects that show ODMR is as follows: Large confocal scans are performed of the sample. A typical confocal scan of a ~5 x 5 μm region is shown in Fig. S1. From this confocal scan, the coordinates of emitters that meet brightness and size requirements (typically a signal-to-noise ratio of 2.5 and diameter of 300-500 nm) are selected. ODMR scans (see below for more experimental details) are performed (between 1.8 and 2.1 GHz) in the absence of applied magnetic field at 100-200 μW laser power (532 nm excitation) with microwave power of ~35 dbm (3 W) into the antenna for ~ 5 mins. Following this approach, we obtain a yield of ODMR-active defects between 1-10%, depending on sample and measurement conditions.

In Figure S2 a 100 x 100 μm region of the sample is shown, where green circles mark the presence of ODMR-active defects.

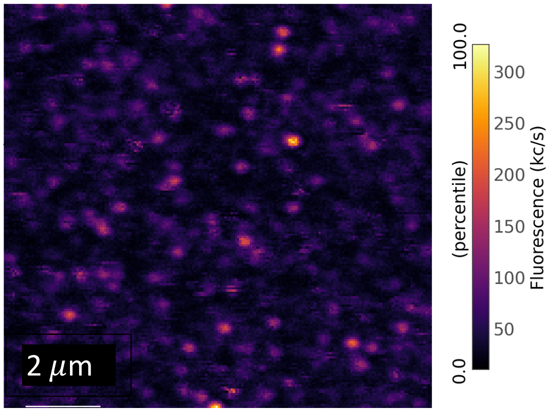

Figure S1: Confocal scan of hBN. *The photoluminescence count rate for the defects ranges from 50 -500 x10^3 counts per second (c/s) with 100 μW of 532-nm illumination.*

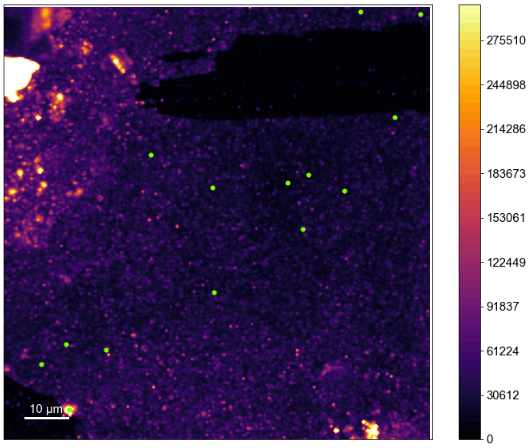

Figure S2: Confocal scan of a 100 x 100 μm region of the hBN sample*. The colour bar is in counts per second (c/s) and green circles show the position of ODMR-active defects. Dark regions are the Si/SiO$_2$ substrate, very bright regions are thicker hBN.*

1.2 Second-order intensity-correlation ($g^{(2)}(t)$) measurements.

We perform second-order intensity-correlation ($g^{(2)}(t)$) measurements to confirm single photon emission from the ODMR-active defects, via the same method as discussed in *(2)*. Briefly, we performed the $g^{(2)}(t)$ measurements by measuring the photon arrival time at two detectors using a Hanbury-Brown-Twiss setup. The recorded arrival times at detector 1 were compared with the arrival times recorded at detector 2, to form an autocorrelation that extends to 1 millisecond separation time. For the data we present, the time stamps from -50 ns to 50 ns are binned in linear-scale time bins, whereas the data at longer time delays are binned in logarithmic-scale bins, to aid visualisation. We used no additional spectral filtering or background correction in the measurement or analysis of the data.

The time-binned data for six ODMR-active defects, at a range of optical pumping saturation parameters, is shown in Fig. S3 (purple circles), with the corresponding PL spectrum for the same defect (where this was measured) shown in the inset. The data is fit to a linear combination of exponential functions (either two- or three-component fits) to functions of the form:

$$f(t) \ = 1 - A\, e^{-\frac{|t|}{\tau_{AB}}} + B\, e^{-\frac{|t|}{\tau_B}}$$

(Eq. S1)

$$f(t) = 1 - A\, e^{-\frac{|t|}{\tau_{AB}}} + B\, e^{-\frac{|t|}{\tau_{B1}}} + C\, e^{-\frac{|t|}{\tau_{B2}}},$$

(Eq. S2)

where A, B, and C are coefficients, τ_{AB} is the antibunching timescale and τ_{Bi} are the bunching timescales (*i*= 1,2). We identify that most of the defects we study require a three-component function to fit the data, however some do not (*i.e.* (b) in Table S1). From the fit we determine the fitted value of $g^{(2)}(0)$, before any background correction, and compare this to the $g^{(2)}(0)$ given by the data, and both values are presented in the inset of Fig. S3. We use the antibunching lifetime we determine via the $g^{(2)}(t)$ measurements to infer the optical lifetime for the defects. The antibunching lifetime is related to both the optical pumping rate and radiative decay rate between the optically excited electronic states and ground states($\frac{1}{\tau_{AB}} = \frac{1}{k_{opt}} + \frac{1}{k_{rad}}$), therefore the y-intercept of a linear fit to $\frac{1}{\tau_{AB}}$ gives an estimation of the optical lifetime *(3)*. The laser power dependent antibunching times for a selection of defects from Fig. S3 are shown in Fig. S4. We determine a radiative lifetime of 5- 6 ns.

Table S1: The fit parameters for $g^{(2)}(t)$ measurements acquired at 50-100 μW (the laser power used for ODMR measurements) for the defects shown in Fig. S3. Some of the measurements are best fit using two decay timescales (one bunching timescale) but most require three decay timescales (two bunching timescales), as observed for ODMR-active defects in hBN previously *(2)*.

Defect label (Fig S3)	A	τ_{AB} (ns)	B	τ_{B1} (ns)	C	τ_{B2} (ns)
(a)	0.87 ± 0.03	5.8 ± 0.3	0.030 ± 0.005	$(2.0 \pm 1.8) \times 10^4$		
(b)	0.68 ± 0.04	4.81 ± 0.48	0.057 ± 0.01	$1.38 \pm 0.93 \times 10^4$		
(c)	0.80 ± 0.03	5.4 ± 0.3	0.10 ± 0.01	$1.18 \pm 0.44 \times 10^5$	0.08 ± 0.01	$3.48 \pm 1.6 \times 10^3$
(d)	0.98 ± 0.03	4.88 ± 0.27	0.06 ± 0.04	$7.78 \pm 7.96 \times 10^4$	0.07 ± 0.04	$6.81 \pm 6.04 \times 10^3$
(e)	1.24 ± 0.04	5.15 ± 0.26	0.48 ± 0.01	$7.22 \pm 0.61 \times 10^5$		

**(f)**	0.99 ± 0.04	4.63 ± 0.31	0.09 ± 0.04	1.248 ± 0.92 x 10^5	0.297 ± 0.05	1.121 ± 0.28 x 10^4
**(g)**	0.66 ± 0.03	4.70 ± 0.33	0.050 ± 0.005	6.22 ± 3.31 x 10^4		
**(h)**	0.87 ± 0.03	5.54 ± 0.32	0.077 ± 0.004	1.66 ± 0.61 x 10^5		

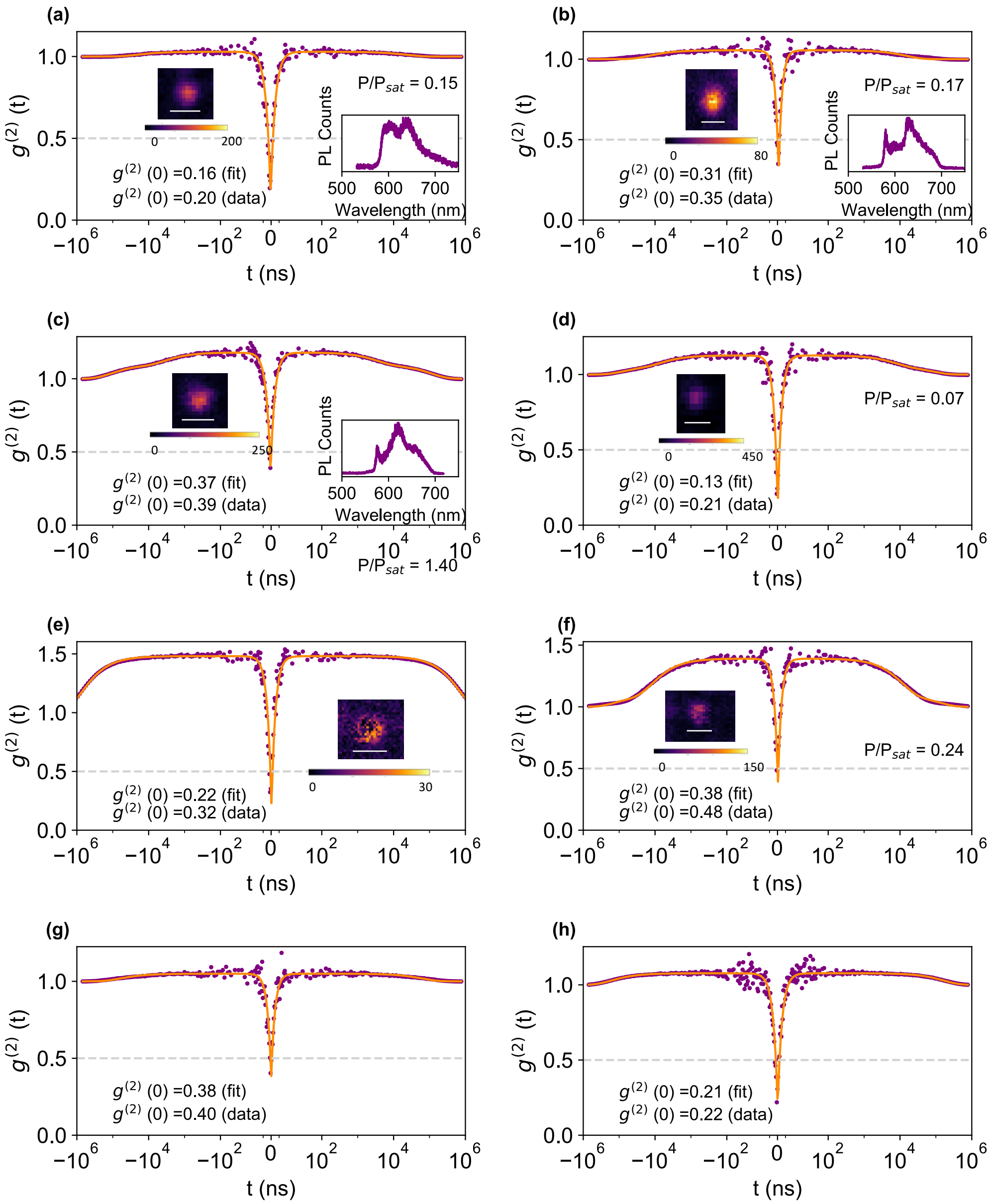

Figure S3: $g^{(2)}(\tau)$ measurements for six ODMR-active defects we study*. We fit the data (purple circles) to either a biexponential or a triexponential function given by Eq. S1 and S2 (orange curves). The insets show the photoluminescence spectra (where this was measured) at room temperature using 20-second acquisition time and a 550 nm long pass filter, and confocal scan of the defect (the white scale bar represents 500 nm and z scale is 10^3 counts per second (kc/s)). The inset text notes the value of $g^{(2)}(0)$ obtained from the data and the fit (before any background correction), as well as the laser power relative to the saturation laser power (P/P$_{sat}$) that the measurement was performed at.*

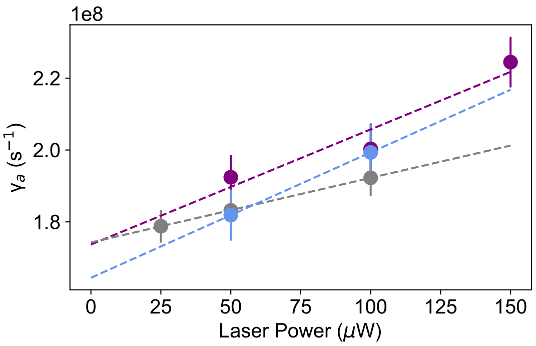

***Fig S4: Estimation of the radiative lifetime**. Antibunching rates as a function of laser power for three defects. The data presented is the fitted value to a single $g^{(2)}(t)$ measurement, using Eqs. 1 and 2 above. Error bars indicate a 68% confidence interval. The antibunching lifetime is determined by both the optical pumping rate and radiative decay rate between the optically excited electronic states and ground states(* $\frac{1}{\tau_{AB}} = \gamma_{AB}$ *and* $\frac{1}{\tau_{AB}} = \frac{1}{k_{opt}} + \frac{1}{k_{rad}}$*), therefore the y-intercept of a linear fit to* $1/\tau_{AB}$ *gives an estimation of the optical lifetime (3). The three defects give* τ_{rad} *= 5.75(0.3), 5.72(0.3) and 6.08(0.5) ns.*

1.3 Integrated photoluminescence (PL) spectra for ODMR-active defects.

The integrated PL spectra for 20 ODMR-active defects are presented in Figure S5. Each defect was illuminated with CW 532 nm light. As can be seen, the defect spectra show a zero-phonon line (ZPL) accompanied by structured phonon side bands (PSBs) of varying intensity and linewidth. To compare the ZPL energy and side-band splitting, we fit the spectra to a sum of four Lorentzians, as well as a non-zero background. We find that four Lorentzians provide an adequate fit to the PL spectra in most cases, but not all. The sum of the four Lorentzians is plotted in red.

A histogram of the ZPL energies for the ODMR-active defects is presented in Fig. S6(a). We find that the 20 ODMR-active defects all display ZPL energies between 2.0 and 2.2 eV, a relatively narrow distribution compared to the range of ZPL energies reported for visible single-photon emitting defects in hBN *(4,5)*. We note that our measurements are conducted with a long pass of 550 nm (2.25 eV) and our optical collection fibre (Thorlabs SM630HP) is optimal in the range between 600 and 770 nm (2.07 -1.61 eV), so our experiments are most sensitive to excitonic emission in the range 2.25-1.61 eV.

In addition to the distribution of ZPL energy, our analysis shows that, for the ODMR active defects, emission into the PSB arises predominantly from coupling to phonon modes with energies ~50-60 meV and ~150-160 meV (Figure S6(b)). Phonon energies in this range have been predicted for carbon-based hBN defects and are respectively assigned to intralayer breathing and stretching modes of the hBN lattice *(6-8)*.

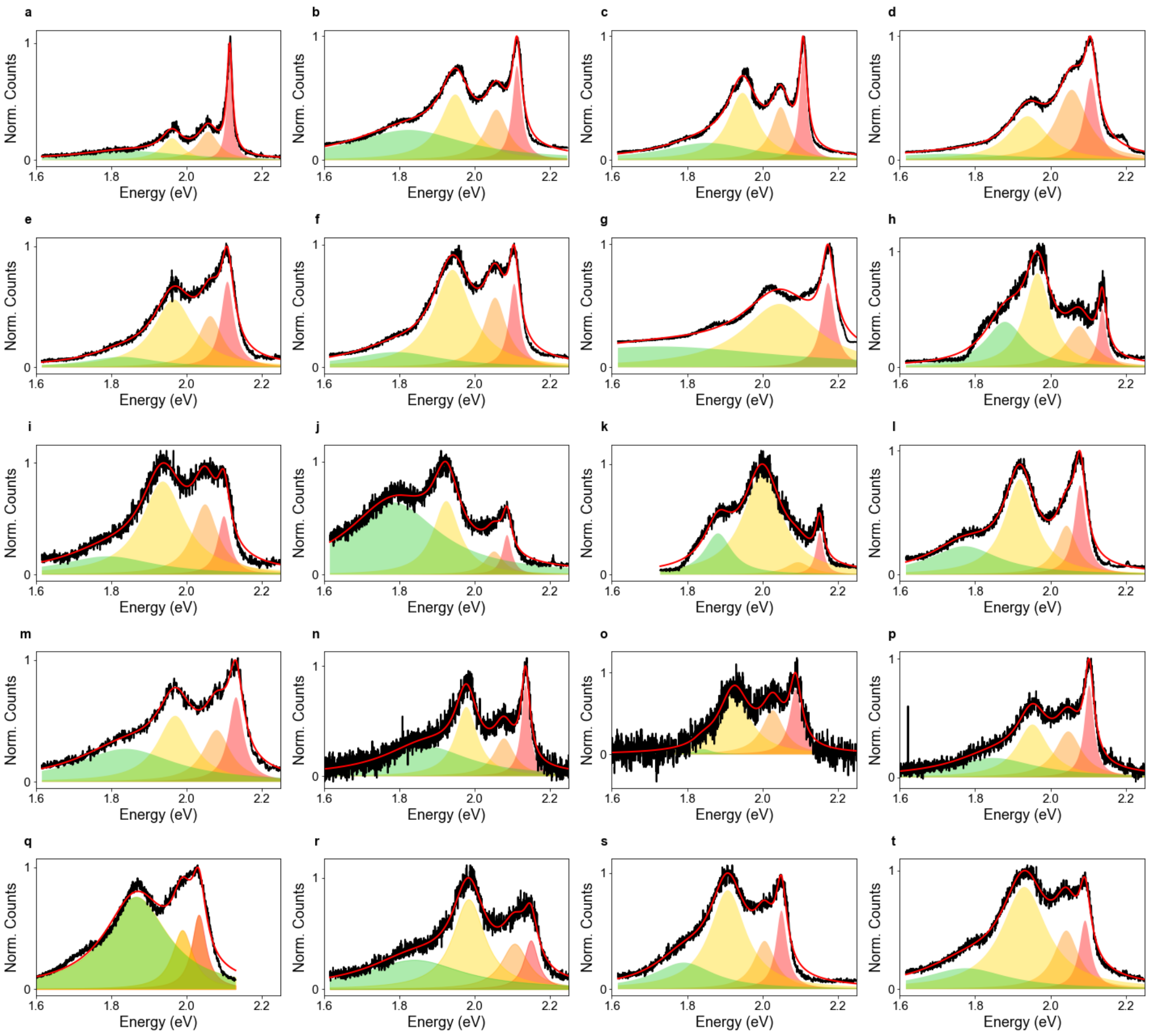

Figure S5: *Integrated PL spectra of ODMR-active defects. Each spectrum is fit to a sum of four Lorentzians and a non-zero flat background. The shaded areas show the individual Lorentzian fits and the red curve shows the overall fit.*

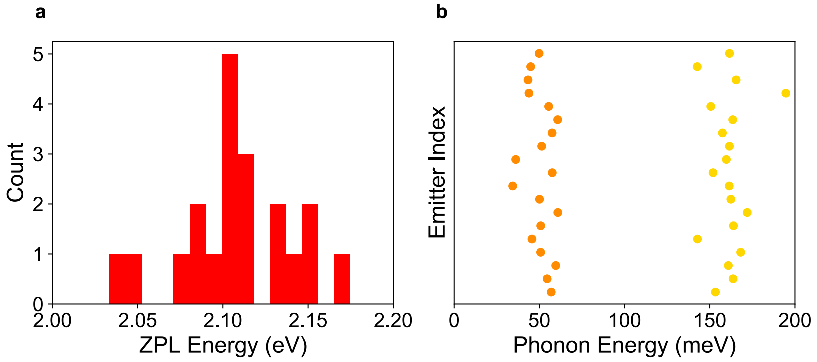

Figure S6*: (a) Distribution of the ZPL energy of the 20 defects obtained from the fits in Figure S5. The energy is extracted from the central frequency of the Lorentzian fit to the high-energy peak. (b) The energy separation between the high-energy peak (ZPL) and the two lower-energy peaks (the PSBs). The y axis is the defect count.*

2. ODMR measurements

2.1 Determination of microwave and optical saturation conditions.

To determine the saturated contrast and unsaturated linewidth of the ODMR spectra we perform laser-power dependent and microwave-power dependent measurements of the defects. Figure S7 shows this data for the same defect.

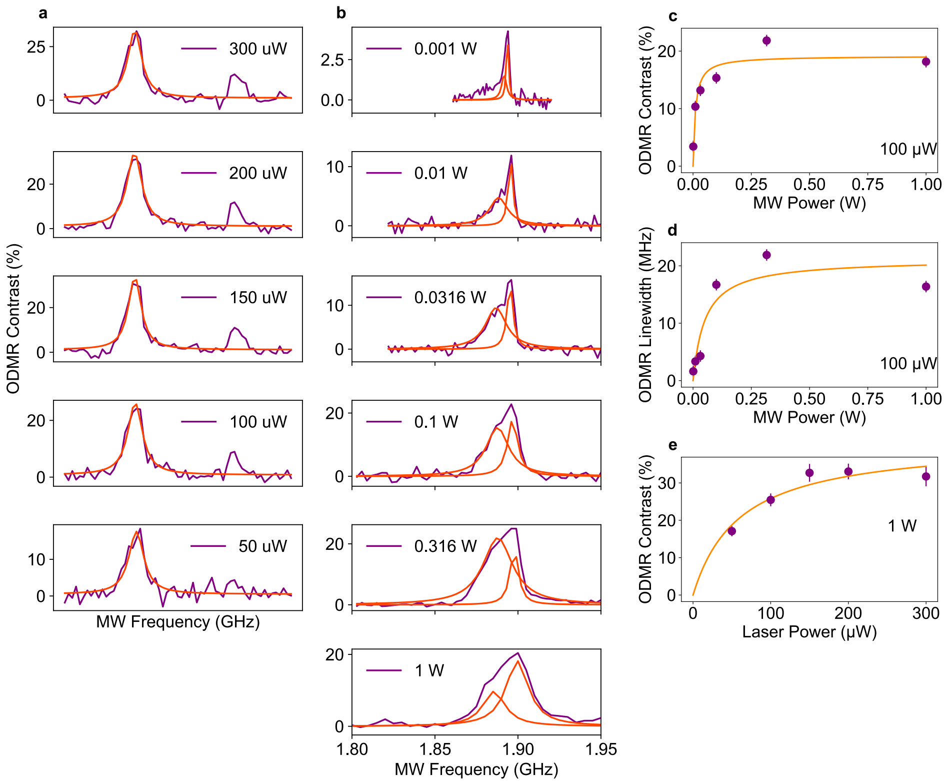

Figure S7: Microwave and laser power saturation of ODMR at 0 mT. *(a) ODMR spectra measured at different laser powers, at 1W microwave power. (b) Microwave-power dependence (0.001-1W) at 100 µW laser power. The data in (a) (purple traces) are fit to a single Lorentzian (red curves) to obtain the peak contrast. The data in (b) is fit to a double Lorentzian. (c) Microwave-power dependence of ODMR contrast, obtained from the higher frequency Lorentzian. (d) linewidth at 100 µW laser power. (e) Laser power dependence of ODMR contrast, at 1W microwave power. All data (purple circles) are fit to* $C = C_{sat}P/P+P_{sat}$ *where C is the ODMR contrast/linewidth, P is the microwave/laser power,* C_{sat} *is the ODMR contrast/linewidth at saturation and* P_{sat} *is the saturation laser power. The data presented in (c,d and e) are the extracted values from fits shown in (a) and (b). Error bars indicate the 68% confidence interval.*

2.2 Observation of the previously reported ODMR resonances

The ODMR resonance presented in our current manuscript presents unambiguously a distinct spin resonance than that presented in references *(2)* and *(9)*, due to the presence of a 1.96 GHz zero-field splitting. In contrast, both previous reports showed that an applied magnetic field was required to observe ODMR, leading to assignment of S=1/2 and S=1 or S=3/2 with low ZFS respectively. This key difference indicates we are studying a distinct electronic state from both previous reports.

In *(2)*, an ODMR resonance that was not present in the absence of a magnetic field was identified on the same sample that we study here. In *(2)* this was assigned to a $S > ½$ resonance with low zero-field splitting due to the presence of fine structure. We can confirm that some individual defects display simultaneously the ODMR resonance reported in the current work (with a clear 1.96 GHz zero-field splitting) and the resonance treated in *(2)* (Figs. S8-10).

We unsuccessfully tried to account for all spin-resonances considering a single spin model (for instance, S=3/2) (see Section 4.1). Instead, a S=1 model accurately reproduces the magnetic-field dependence of the magnetic resonance we study in this work. Consequently, we postulate that the two distinct spin resonances relate to different electronic states of the same defect (either optical or charge states). One possibility is that the resonance presented in this work corresponds to the electronic ground state (1.96 GHz zero-field splitting) whereas the resonance studied in *(2)* arises in the optically excited state (<50 MHz zero-field splitting). Interestingly, we find that not all defects that display the 1.96 GHz ZFS transition also show the 50 MHz ZFS transition.

An alternative possibility is that the two spin signatures correspond to different charge states that show different spin resonances, *i.e.* interconversion between S=1 and S=3/2 states where the S=3/2 ground state has a spin resonance with low zero-field splitting. Depending on the timescale of conversion (if this is relatively fast), both spin resonances may be measured via cw-ODMR at the same time.

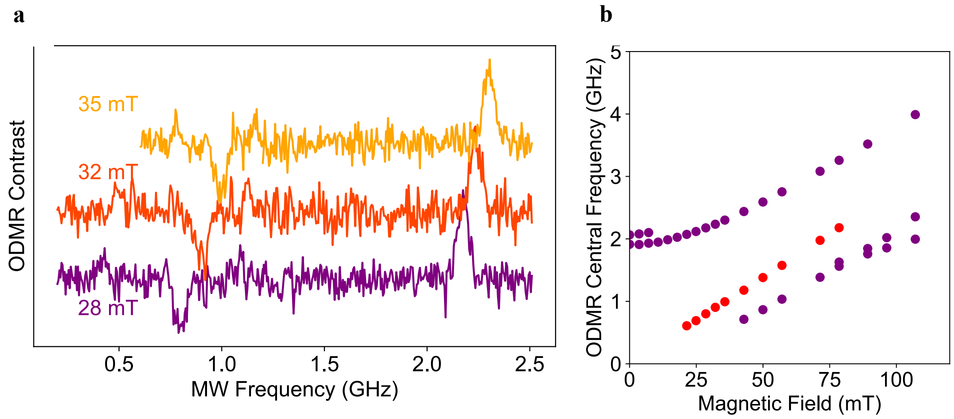

Figure S8: Observation of multiple ODMR resonances for a single defect*. (a) ODMR spectra for a single defect showing the low frequency, previously identified (2) resonance (~ 800 MHz) and high frequency ground-state resonance studied in this report (~ 2.2-2.4 GHz), at an applied, off-axis magnetic field of 28- 35 mT. This is the defect shown in Figure S3(c). (b) The ODMR resonance frequencies measured for a defect that shows both resonances. The purple circles represent the transition energies of the S =1 ground states (including the* $|+\rangle$ *to* $|-\rangle$ *transition at magnetic field above 40 mT) and the red circles represent the ODMR transition energies of the previously identified resonance. This is the defect shown in Fig. S3(c).*

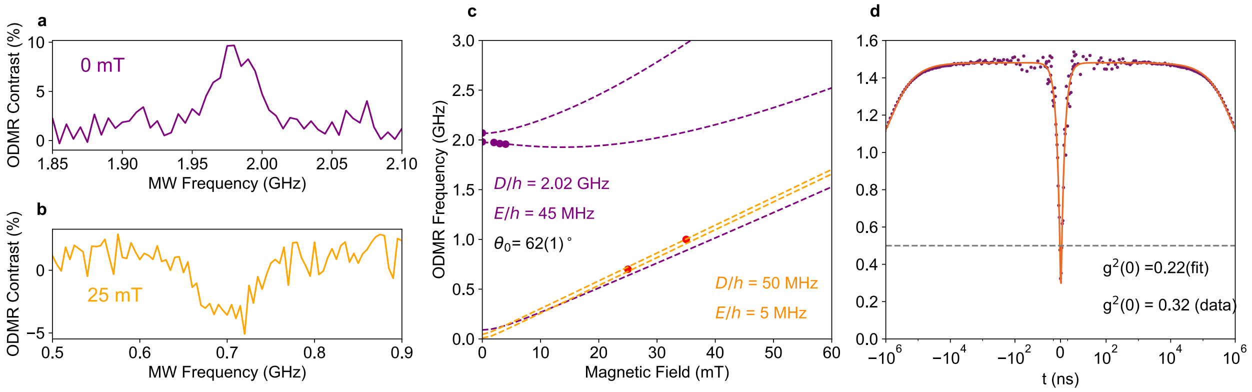

Figure S9: Observation of multiple ODMR resonances for a single defect *(a) ODMR at 0 mT (b) ODMR at 25 mT (c) Experimentally observed ODMR resonances (circles) with fits to S=1 model (purple and orange lines) (D/h and E/h parameters shown in inset) where* θ_0 *is the angle between the defect quantisation axis and* ***B****. (d)* $g^{(2)}(\tau)$ *measurement for this emitter showing* $g^{(2)}(0)$ *(fit) in the absence of any background subtraction.*

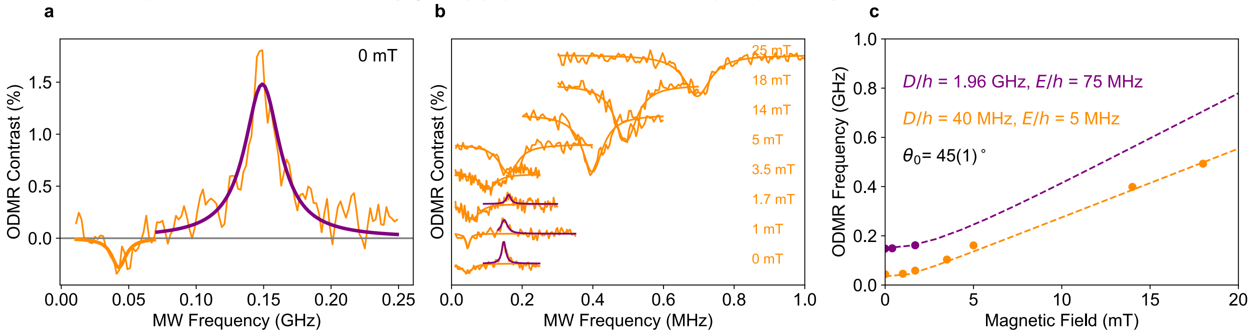

Figure S10: Observation of multiple ODMR resonances for a single defect*: (a) ODMR at 0 mT (b) ODMR spectra obtained with an applied magnetic field of 0-25 mT. Orange curves show fits to negative contrast peak shape. Purple curves show fits to positive contrast peak shape. (c) Central frequency for the data shown in (b). Experimentally observed ODMR resonances (circles) with fits to S=1 model (purple and orange lines) (D/h and E/h parameters shown in inset) where* θ_0 *is the angle between the defect quantisation axis and* ***B****.*

2.3 Magnetic field dependent ODMR

In our setup, we can apply a magnetic field in two ways:

1. We use a permanent magnet mounted on an angular mount that allows us to vary the direction of the magnetic field in the plane defined by the hBN layers, while keeping its magnitude approximately fixed.
2. We use a calibrated magnet fixed to translation stage that allows us to vary the magnitude of the field applied to the defect, giving us access to up to 100-mT applied fields.

Investigating the ODMR resonance under applied magnetic field in these two configurations allows us to confirm the orientation of the defect's *z*-axis relative to the lab frame and to the hBN layers.

For a *S*=1 defect under applied magnetic field, the Hamiltonian is given by:

$$H = g\mu_B \boldsymbol{B}\left(sin\theta_0 cos\phi_0 S_x + sin\theta_0 sin\phi_0 S_y + cos\theta_0 S_z\right) + D\left(S_z^2 - \frac{S(S+1)}{3}\right) + E(S_x^2 - S_y^2), \qquad \textbf{(Eq. 3)}$$

where the $x, \ y, \ z$ axes are defined by the internal structure of the defect, and the angles θ_0 and ϕ_0 between these axes and the applied magnetic are as defined in Fig. S11.

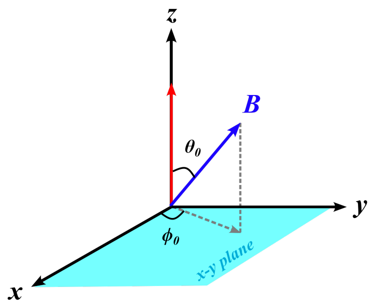

Figure S11: Axes defined in the diagonalised frame of the zero-field tensor. *The principal axis of **D** with principal value proportional to D (red vector) defines the z-axis in this frame. θ_0 is the angle between z and **B** (blue vector), and ϕ_0 is the angle between x and the projected B-vector in the x-y plane.*

To confirm the orientation of the defect's *z*-axis relative to the lab frame we compare the transition energies predicted by the Hamiltonian in Eq. 3 to those extracted from a series of angular- and magnitude-dependent ODMR measurements. Angular-dependent measurements allow us to unequivocally determine the direction of the defect's *z*-axis with respect to the hBN layers, whereas amplitude-dependent measurements allow us to quickly probe in-plane direction the defect's *z*-axis. We compare the orientation of a given defect obtained with either approach in order to extract the accuracy of our measurements.

Angular-dependent ODMR

To confirm the orientation of the defect's *z*-axis relative to the lab frame we vary the magnetic-field orientation while keeping its amplitude constant, according to measurement 1 presented above. To present our results, we must define a new set of axes (x_{lab},y_{lab} and z_{lab}) in the lab-frame, where y_{lab} is the optical axis and the hBN layers lie in the z_{lab} - x_{lab} plane (Fig. S12). The angle between **B** and z_{lab} is θ'. In Figure S13(a) we present υ_1 and υ_2 for the same defect that is presented in Figure 1(b) in the main text as a function of θ' *i.e.,* the magnet is moved in the z_{lab} - x_{lab} plane, with |B| = 14(1) mT. The data is the purple circles and the resonances predicted using an *S*=1 model are shown by the purple curves. We find that the ODMR transition energies are very sensitive to θ', indicating that the defect's *z* axis lies in the hBN plane.

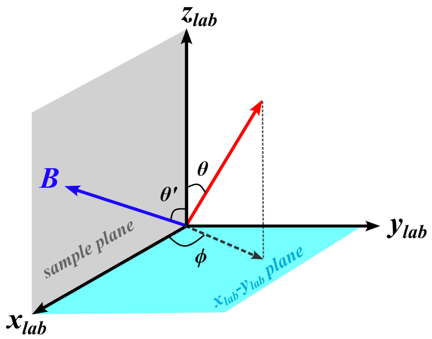

Figure S12: Definition of the lab frame axes. *Definition of the lab frame coordinates (x_{lab},y_{lab} and z_{lab}), where θ' defines the angle between B (blue vector) and z_{lab} , θ and ϕ are the polar and azimuthal angles of the defect axis of highest symmetry (red vector) in the lab frame.*

We convert the orientation of **B** in the lab frame (θ', ϕ') to an orientation with respect to the defect's z-axis, defined by the angles (θ, ϕ). The simulation fits the data with |B| = 14.15 $\pm$ 0.15 mT, θ = 24 $\pm$ 1° and ϕ = 0 $\pm$ 18° which show that the axis of highest symmetry is almost in the plane of the hBN layers (Figure S13(b)). We verify this with ODMR measurements taken at θ' = 24(5)° and varying magnetic field amplitude (Fig. S14). Here, the ODMR transition energies evolve symmetrically with increasing amplitude of the applied field, consistent with the eigenstates of an S = 1 model where **B**||z (fit shown in Fig. 1 of main text).

We compare the orientation of this defect's *z*-axis with respect to the lab frame determined in this way to the results we obtain from the field-amplitude dependence of the ODMR transition energies for the same defects (Fig. S15 (a)). We find a difference of 6° in the orientation of defect *z*-axis relative to the lab frame, determined via these two methods. We consider this difference of ~10° is related to the error in θ' during the rotation, due to the small unavoidable misalignment of the centre of magnet rotation axis relative to the confocal spot. Also, we expect a small change in field strength (~ 1 mT) during the rotation due to this misalignment. Overall, we find the defect *z*-axis, for all the defects where we performed the angular dependence (six), lies in the plane of the sample.

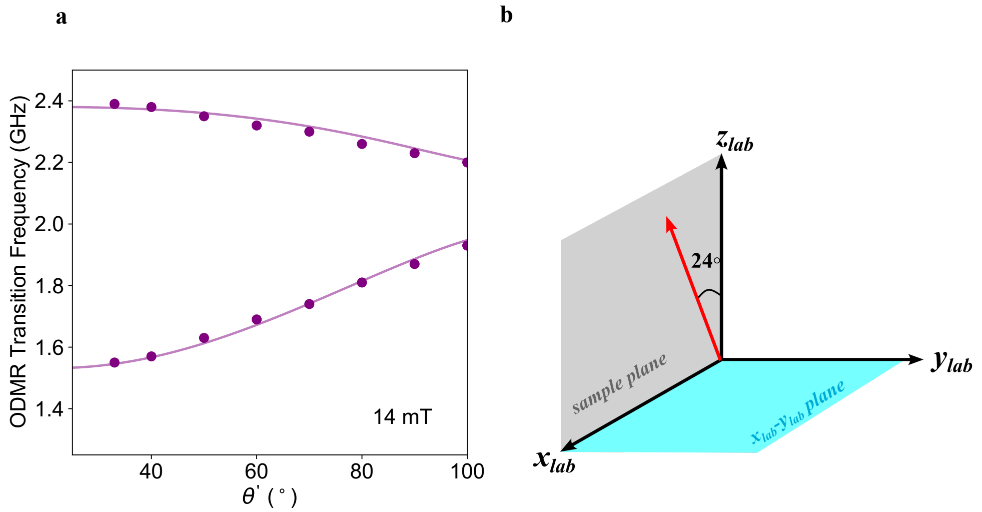

Figure S13: Determination of defect z-axis and resulting defect symmetry in the lab frame. *(a)The ODMR transition energies (circles) and S = 1 model (purple curve) for the angular dependence. (b). The axis of highest symmetry (red vector) lies in the plane of the hBN layers.*

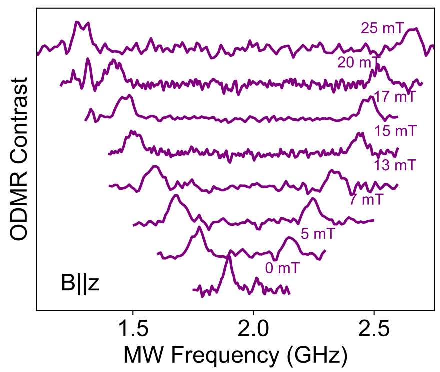

Figure S14**: **On-axis ODMR spectra. *The ODMR spectra measured where θ'= 24(5)° for the defect shown in Fig. S13(a).*

Field-dependent ODMR

Once we identify ODMR-active defects at zero magnetic field, we study the field-magnitude dependence of the ODMR resonances. We perform these measurements using a calibrated magnet that is fixed to translation stage in the x_{lab} direction, providing access to up to 100-mT applied fields along x_{lab}. For most of the defects, we measured up to 40 mT, as this was adequate to get a good fit to the field-amplitude dependence. Our sample is mounted along the x_{lab}-z_{lab} plane: this means the magnetic field is applied along the plane containing the hBN layers, at an arbitrary orientation relative to the z-axis of each defect. Figure S15 shows the evolution of the ODMR transition energies for nine different defects in the same sample, as a function of the amplitude of the applied magnetic field **B** (purple circles). The data is fit to the eigenstates of a $S=1$ spin Hamiltonian, where θ_0 and ϕ_0 are defined as the polar and azimuthal angles of the B-field in the diagonalised frame of the zero-field tensor (Fig. S11). We determine θ_0 for each emitter and note it in the subplot inset. The value of ϕ_0 is indetermined from the field-dependence at a fixed orientation, has little effect on the model and is set to 10°.

Interestingly, for some defects we observe a low-frequency ODMR resonance that we assign to the $|+\rangle$ to $|-\rangle$ transition. This transition is observed when the magnetic field strength is > 40 mT and when **B** is significantly tilted from the orientation of the defect principal axis. We measure that the ODMR contrast is highest with an on-axis **B**. For an off-axis **B**, the contrast drops as the magnetic field strength increases (Fig. S16), although we note that that contrast is not completely quenched and for some of the defects the contrast remains up to high off-axis -field strengths (~100 mT), unlike for NV centres in diamond *(10)*. The retention of ODMR contrast under high off-axis fields is likely to be related to spin-dependent photodynamics and the nature of the spin mixing in this system, which should be further investigated. Importantly, the sensitivity of the ODMR to off-axis fields may mean this defect is well-suited to magnetometry of systems where detection of fields of tens of mT is required.

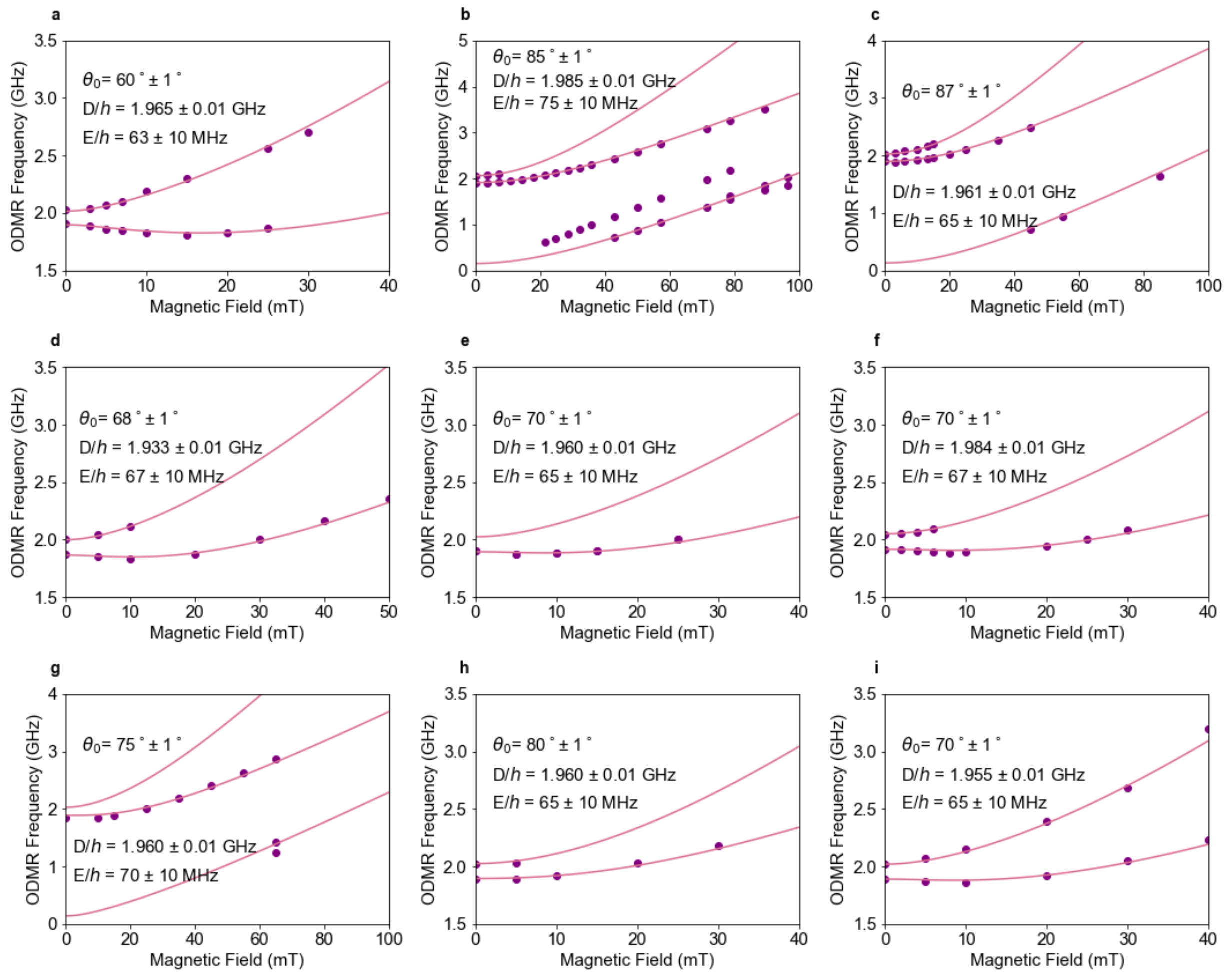

Figure S15: Magnetic field-magnitude dependence of the ODMR transitions. *Field-dependence of the ODMR resonances (purple circles) for nine different defects, conducted with an in-plane magnetic field. The data is fit to an S = 1 model (pink curves). The orientation of the defect z-axis relative to* ***B*** *(θ_0) is determined from the fitting process and is noted in the inset of each figure.*

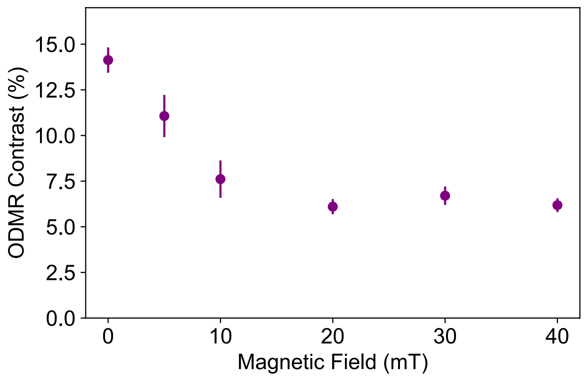

Figure S16: Persistent ODMR contrast with an off-axis field. *Saturated ODMR contrast for a defect ((d) in S15) with an off-axis* ***B*** *(θ_0 = 68(1)°). The data presented are amplitudes extracted from fits of the ODMR spectra at each magnetic field to a Lorentzian curve. Error bars represent the 68% confidence interval of the fit parameter.*

2.4 Zero-field ODMR

We identify ODMR-active defects via automated confocal scanning combined with ODMR at zero magnetic field. Figure S17 plots sample zero-field ODMR-resonances for 30 of the defects we have studied. We fit each resonance of the zero-field ODMR spectrum with a Lorentzian to obtain the zero-field splitting parameters. Across all defects we study we measure D/h = 1.970 ± 0.02 GHz and E/h = 62 ± 7 MHz. The scanning range was constant between 1.7 and 2.1 GHz. The saturated ODMR contrast varies between the defects (1 - 50%).

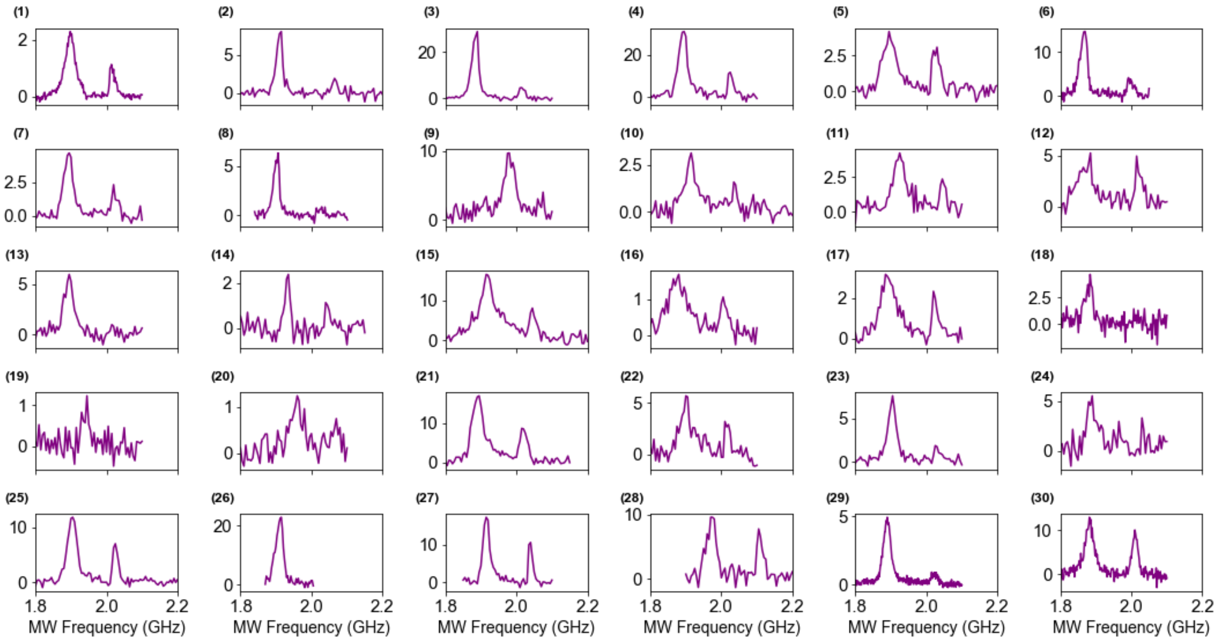

Figure S17: Zero-field ODMR of single defects. *These measurements have been obtained at high microwave power relative to saturation and 100-200 µW laser power. The y axis is ODMR contrast (%).*

3. Coherent microwave control

3.1 Readout calibration and pulse sequences

To perform pulsed ODMR measurements we determine the optimum initialisation and read out duration via a calibration measurement. In this measurement we apply a long green pulse followed by a microwave pulse (50 ns) and a second initialisation pulse accompanied by a 500 ns readout pulse. The readout window is iteratively delayed relative to the start of the initialisation pulse. Figure S18 shows the ODMR contrast measured for this measurement as a function of the readout delay. When the contrast returns to zero, the system is fully initialised, which in this case is ~ 80 μs. We find that most defects require 30-100 μs initilisation with 100 μW of 532 nm, consistent with the bunching dynamics (Fig. S3 and Table S1). The optimum readout duration is determined from analysing the signal to noise (accumulated contrast vs. accumulated shot noise) as a function of readout start and stop time (Fig. S19). Typically, the optimal readout duration is 20-40 μs.

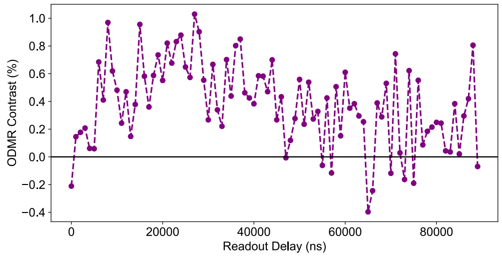

Figure S18: Spin intialisation. *ODMR contrast (%) as the readout window is scanned over the initialisation laser pulse (readout delay (ns)).*

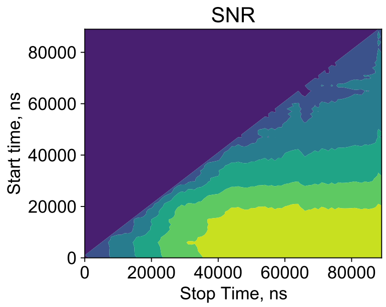

Figure S19: Readout duration dependence of signal-to-noise. *ODMR signal-to-noise determined as a function of readout start and stop time. The colour scale shows the signal-to-noise ratio. In this case, the optimal readout duration is 60 μs.*

3.2 T_1 measurement of the previously studied resonance.

The values for the spin lattice relaxation time that we measure for the ground-state resonance vary from 35 - 200 μs. This is much longer than T_1 measured for single hBN defects previously *(2,11)*. Below we show the T_1 measured for the ODMR resonance that we have previously reported in *(2)*. Here, the T_1 is ~ 9 μs, measured at a field strength of 70 mT.

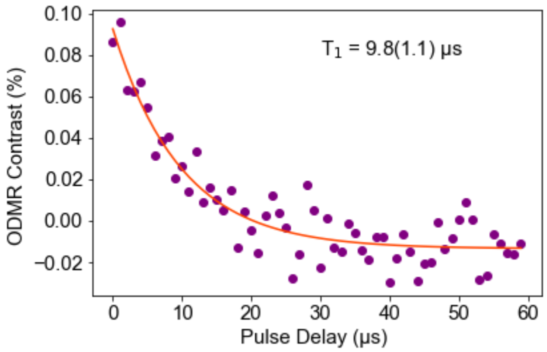

Figure S20: Spin-lattice relaxation of a separate ODMR resonance. *T_1 measurement for the ODMR resonance measured in (2).*

3.3 Rabi measurements

We fit the Rabi oscillations presented in Fig. 2 of the main text to a function of the form,

$$Y(t) = A \exp[-(t/T_{Rabi})]\sin(2\pi\Omega-\varphi)$$

(Eq. S4)

where T_{Rabi} is the decay of the Rabi envelope, Ω is the Rabi frequency of each component of the Rabi and φ is the phase offset. Below is the dependence of the Rabi frequency on the square root of the microwave power. We observe a linear relation, as expected (Fig. S21).

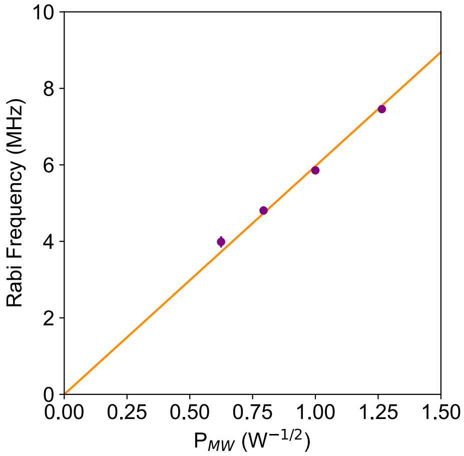

Figure S21: Rabi frequency microwave power dependence. *Rabi frequency as a function of the square root of the microwave power ($W^{-1/2}$). The circles are the fitted Rabi frequency (using Eq. S4) to a single measurement at each microwave power. Error bars indicate the 68% confidence interval.*

3.4 Pulsed ODMR lineshape

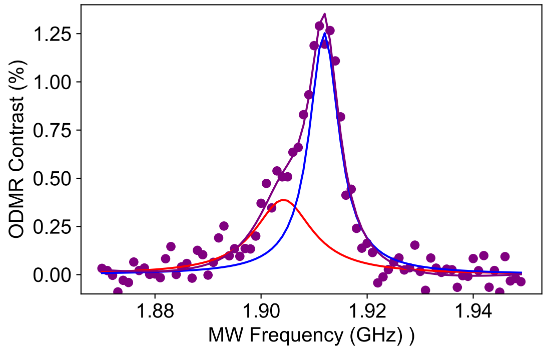

***Figure S22: Unsaturated Pulsed ODMR spectrum**. We fit the data to two Lorentzians, labelled ν_{1b} and ν_{1a}. The peak separation is 10 MHz and respective linewidths are 13 MHz and 6 MHz.*

4. Spin Hamiltonian

4.1 Electronic spin Hamiltonian model and *S*=3/2

In the main text we model the magnetic-field dependence of the ODMR spectra based on an effective-spin Hamiltonian composed of zero-field and Zeeman terms. The observation of zero-field ODMR resonances requires a spin-Hamiltonian with multiplicity > 2 (that is, an effective-spin system $S \geq 1$). We consider both S = 1 and S = 3/2 possibilities but find that S = 3/2 is inconsistent with our data, as discussed below. Larger effective-spin models are highly unlikely since they would require electronic wavefunctions containing more than 4 unpaired electron spins, or eigenstates with large orbital angular momenta.

An S = 3/2 system may arise for a wavefunction with three unpaired electron spins. In this case, the exchange interaction between the electrons causes a splitting between two Kramers doublets at zero magnetic field. The zero field ODMR spectrum would show a single zero-field ODMR line at an energy given by $\nu = (\sqrt{D^2 + 3E^2})/h$. We routinely measure a doublet at zero field, so S = 3/2 is unlikely.

In the high-field regime (when the Zeeman interaction is much larger than the zero-field splitting between the levels), an S = 3/2 system gives rise to two ODMR-active transitions that are equally spaced and evolve linearly with magnetic field amplitude. Importantly, the two ODMR transitions are equally spaced from the position of a free S = 1/2 resonance. This is not what we measure for our defects (see Fig. S15). Furthermore, a S = 3/2 system cannot explain the low frequency $|+\rangle$ to $|-\rangle$ transition that is observed in our measurements.

Finally, a wavefunction with three unpaired spins may arise in systems with cubic symmetries, where the high symmetry allows an orbital triplet to appear. However, such high symmetry is impossible in a two-dimensional system. Alternatively, the unlikely scenario where the system shows accidental degeneracy between an orbital doublet and an orbital singlet may lead to a wavefunction with three unpaired spins. Nonetheless, in total, the qualitative description of the magnetic-field dependence of the ODMR spectrum of an effective-spin 3/2 system fails to encompass the behavior we measure for the emitters presented in this work, and we discard the possibility that we are dealing with a spin-3/2 system.

4.2 Hyperfine models

Low-field behaviour.
We can understand the ODMR spectra lineshape in the few-mT regime if we assume that the lineshape of the ODMR transitions is governed by hyperfine interaction with the nuclear-spin bath surrounding each defect.

At zero field, the transverse zero-field splitting parameter *E* gives rise to an anticrossing related to a clock transition. In this regime, the eigenstates of the system are given by $|0\rangle = |0_z\rangle$, $|\pm\rangle = \frac{1}{\sqrt{2}}(|+1_z\rangle \pm |-1_z\rangle)$. These eigenstates have zero expectation value for the electronic spin projection along any axis ($\langle S_x\rangle = \langle S_y\rangle = \langle S_z\rangle = 0$), quenching the hyperfine coupling between the electronic wavefunction and neighboring nuclear spins. This is consistent with the broadening of the ODMR linewidth upon application of a small magnetic field (Fig. 4 main text), and the dampening of the Rabi oscillations with an applied field (as discussed in the main text). Clock transitions are observed for other calculated *S*=1 lattice defects in hBN *(12)*, bulk semiconductor materials *(13)* and molecular spins *(14)*.

Finally, the asymmetry in the zero-field lineshape arises due to the hyperfine coupling to neighboring nuclei being comparable to the transverse zero-field splitting parameter *E/h* (60-70 MHz), leading to perturbative mixing between the eigenstates $|+\rangle$ and $|-\rangle$.

High-field behaviour
We attempt to model the ODMR spectrum lineshape at an applied field along the defect *z*-axis by considering the effective-spin Hamiltonian of Eq. 1 of the main text, modified to include the interaction with the nearest neighbouring spins

$$H = H_e + \sum_i \vec{S} \cdot \boldsymbol{A}_i \cdot \vec{I_i} + \gamma_{n,i}\, \vec{B} \cdot \vec{I_i} \qquad \textbf{(Eq. S5)}$$

where $\vec{I_i}$ is the nuclear-spin operator associated with the i-th nuclear spin, $\boldsymbol{A}_i$ is the hyperfine coupling tensor describing the coupling between the i-th nuclear spin and the electronic spin,$\gamma_{n,i}$ is the gyromagnetic ratio of the i-th nuclear spin, and H_e is the Hamiltonian presented in Eq. 1 of the main text. For simplicity, we consider only Fermi-contact interaction terms. This means that we assume that the only non-zero terms in the hyperfine tensor $\boldsymbol{A}_i$ are $A_{i,xx}$, $A_{i,yy}$ and $A_{i,zz}$. When the magnetic field is parallel to the symmetry axis of the defect, the hyperfine coupling terms $A_{i,xx}$, $A_{i,yy}$ act only perturbatively, such that within the secular approximation we only need to consider $A_{i,zz}$ terms. To model the lineshape of the ODMR spectrum, we calculate the eigenlevels E_k and hyperfine-coupled eigenstates $|\psi_k\rangle$ of the Hamiltonian in Eq. S5, when $\vec{B}_0$ has a magnitude of approximately 20 mT and points in the direction parallel to the *z*-axis of the defect. We then use these eigenvalues and eigenvectors to simulate the ODMR spectrum by adding Lorentzians centered at the energies $E_{kj} = E_k - E_j$ with relative amplitudes given by $P_{kj} = \left|\langle\psi_k|\gamma_e\, \vec{B}_1 \cdot \vec{S}\, |\psi_j\rangle\right|^2$, where $\vec{B}_1$ is an oscillating field pointing in the out-of-plane direction with respect to the hBN layers. We assume that the lineshape associated with each individual transition is Lorentzian, with a linewidth of approximately 7 MHz in line with the zero-field pODMR linewidth (Fig. S22). Finally, we assume that nuclear-spin sublevels are all equally occupied. Using these assumptions, the simplest model that qualitatively describes the lineshape we observe when a magnetic field is applied parallel to the symmetry axis of the defect corresponds to a central electronic spin coupled to 2 inequivalent neighbouring nuclear spins. Figure S23 shows that, based on this model, our data is not sufficient to distinguish between the cases where the central electronic spin is coupled to one B and one N, two site-inequivalent B or two site-inequivalent N. The text in the insets of the figure indicates the magnitude of $A_{i,zz}$ used to obtain the lineshape simulations.

Figure S24 shows the calculated ODMR spectra for alternative configurations comprising a defect coupled to n equivalent nitrogen (top row) or boron (bottom row) nuclear spins, with n =1-5. Amongst these configurations, we could not identify a situation that reproduces our data well.

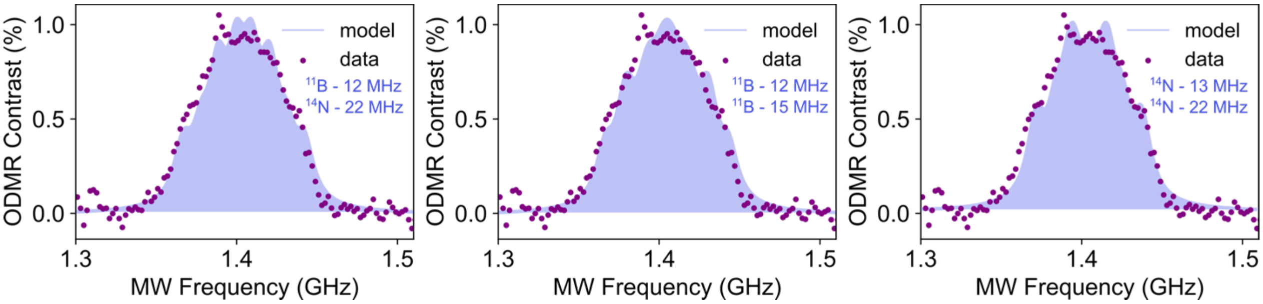

Figure S23: Modeled ODMR lineshape for three models considering coupling between the central electronic spin and two inequivalent neighbouring nuclei. *Within the resolution of our data, we cannot distinguish between the situations where the central spin is coupled to two inequivalent nuclei of different types (one boron and one nitrogen, left panel) or of the same type (two boron, central panel, or two nitrogen, right panel). Nonetheless, this is the only model that captures the ODMR lineshape in the presence of a magnetic field parallel to the symmetry axis of the defect.*

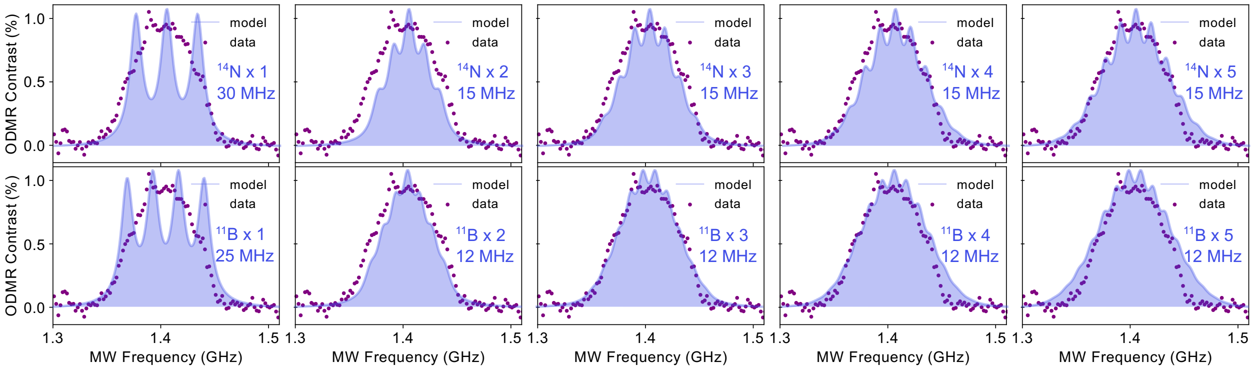

Figure S24: Modeled ODMR lineshape for various nearest-nuclear spin configurations for an electronic spin-1 system. *The top row shows the simulation when the electronic spin is coupled to n equivalent nitrogen-15 (top row) or boron-11 (bottom row) nuclei, with hyperfine coupling on the order of 15 MHz. From left to right, n runs from 1 to 5. For these simulations, we assume a 20 mT magnetic field along the z-axis of the defect electronic spin.*

4.3 Comments on Potential Chemical Structures

We conclude from previous experimental studies *(15)* that the defect structure is likely to, but not definitively, contain carbon. Our ODMR data provide additional important information that relates to the chemical structure of the defect we are measuring.

Our data shows that the defect forms a low symmetry structure. The assignment of low symmetry arises directly from the in-plane spin quantisation axis that we measure for the defect, as well as the transverse zero field splitting parameter *E/h* ~ 70 MHz. Non-zero *E/h* is only symmetry-allowed in structures with symmetry lower than C_3 (that is, in structures where the maximum axial rotational symmetry present is a two-fold rotation). This excludes several candidates from the pool of possible defect structures that could rise to this resonance: for instance, we can rule out simple vacancies

(V_N, V_B), single carbon substituents (C_N, C_B) or four-carbon structures ($C_B(CN)_3$, $C_N(CN)_3$) *(16),* due to these structures being high symmetry.

In addition, we observe hyperfine signatures that are indicative of hyperfine coupling of <20 MHz (Section 4.2). This magnitude of hyperfine indicates a weaker interaction than that expected for a localized defect structure coupled to nuclei one lattice site away (for example in V_B^-, 47 MHz coupling to ^{14}N *(17)*). Instead, this magnitude of hyperfine coupling could be expected for a more delocalized electronic wavefunction in a more extended carbon structure. This weak hyperfine coupling also indicates that the electronic wavefunction is mostly localized on lattice sites occupied by nuclei without a nuclear spin – as co-localization of the nuclear spin and the electronic wavefunction would lead to much larger values of hyperfine coupling. This is consistent with a carbon structure (where ^{12}C is I=0 and is 99% abundant). In addition, the magnitude of D/h suggests a structure that extends over multiple lattice sites.

Regarding specific potential carbon-based structures, we consider it unlikely that the spin resonance we study is due to a carbon dimer because this is predicted to emit ~ 4.1 eV *(6,7,18)*. Carbon trimers (C_2C_N and C_2C_B) are low symmetry and predicted to emit ~ 2eV *(6,8,19,20)*, consistent with what we measure. However, the ground state is expected to be S=1/2 *(6,19)* and thereby incompatible with our findings, while positive and negative charge states of the trimers are predicted to have higher energy optical transitions (3 eV) *(6)*. Vacancy-substituent defects (V_NC_B or V_BC_N) are predicted to have spin-triplet ground states *(16,21)* but the zero-field splitting is predicted to be > 7 GHz in both cases due to the highly localized electronic wavefunction *(16)*. For separated carbon dimers (donor-acceptor pair defects (DAP)), the ZPL energy is predicted to decrease with carbon-carbon separation *(19)* with ZPL and PSB energies that may be consistent with what we measure. However, DAP structures have been predicted to be S=1/2 *(19)*.

5. Appendix

The data presented in the main text is collected from five of the 40 different defects we have studied. Data from these five and other defects are presented in the SI. After studying many defects, we can confirm that the important phenomena we present are largely independent of the defect studied. Apart from the magnitude of the ODMR contrast, all defects respond similarly to an applied magnetic field, *i.e.* both ODMR spectra and the coherence timescales respond similarly to a magnetic field, when the intrinsic direction of the defect axis is taken into account. During our extended study of this system different experiments were performed on different defects. Unfortunately, not all experiments could be measured on a single defect due to defects stochastically going dark after illumination for several weeks/months.

Table S1: List of defects presented in main text and Supplementary figures.

	**Main Text**	**SI**
Defect 1	1(a)	
Defect 2	1(b) and 1(b) inset, 2(c), 4(c), 4(e) and 4(f)	3(a), 5(i), 12, 13, 15(a), 17(1)
Defect 3	1(f), 4(a)	3(c), 5(k), 8, 15(b), 17(2), 18, 19, 22
Defect 4	2(a), 3	7, 15(i), 17(4)
Defect 5	2(b), 4(d)	3(b), 5(h), 15(c),17(5), 21
Defect 6		3(f), 15(h), 17(3)

Table S2: Single hBN-defect spin reports

Sample type	Report	E_{ZPL} (eV)	ZFS (GHz)	Spin multiplicity (S)	Temperature (T)
Carbon doped MOVPE-grown hBN layers	Mendelson *et al.* (ensemble) *(15)*	2.0-2.2	-	> ½	RT
	Stern *et al* (singles) *(2)*	2.0-2.2	<0.1	> ½	RT
	Stern *et al* (singles) (this work)	2.0-2.2	1.96	1	RT
Exfoliated hBN flakes	Chejanovsky *et al.* (singles) *(9)*	1.65-1.77	-	½	5 K
Suspended hBN nanopowder	Guo *et al.* (singles) *(11)*	2.3	-	½	RT

6. References

[1] Binder, J. M., et al. Qudi: A modular python suite for experimental control and data processing. *Original Sotfware Publication*, **6**, 85 (2017).

[2] Stern, H. L., et al. Room-temperature optically detected magnetic resonance of single defects in hexagonal boron nitride. *Nat. Commun.,* **13**, 618 (2022).

[3] Berthel, M., et al. Photophysics of single nitrogen-vacancy centers in diamond nanocrystals. *Phys. Rev. B.*, **91**, 035308, (2015).

[4] Wigger, D. et al., Phonon-assisted emission and absorption of individual color centers in hexagonal boron nitride, *2D Materials*, **6** (2019).

[5] Tran, T. et al., Robust Multicolor Single Photon Emission from Point Defects in Hexagonal Boron Nitride, *ACS Nano.*, **10**, (2016).

[6] Jara, C. et al., First-principles identification of single photon emitters based on carbon clusters in hexagonal boron nitride. *J. Phys. Chem. A.*, **125**, 1325 (2021).

[7] Winter, M., Bousquet, M. H. E., Jacquemin, D., Duchemin, I. and Blase, X. Photoluminescent properties of the carbon-dimer defect in hexagonal boron-nitride: A many body finite-size cluster approach. *Phys. Rev. Mats*., **5**, 095201 (2021).

[8] Li, K., Smart, T. J., and Ping, Y. Carbon trimer as 2 eV single-photon emitter candidate in hexagonal boron nitride: A first principles study. *Phys. Rev. Mat*., **6**, 042202 (2022).

[9] Chejanovsky, N., et al. Single-spin resonance in a van der Waals embedded paramagnetic defect. *Nat. Mater.,* **20**, 1079 (2021).

[10] Tetienne, J-P., et al. Magnetic-field-dependent photodynamics of single NV defects in diamond: an application to qualitative all-optical magnetic imaging, *New J. Phys.*, **14**, 103033 (2012).

[11] Guo, N-J., et al. Coherent control of an ultrabright single spin in hexagonal boron nitride at room temperature. *Nat. Commun.*, **14**, 2893 (2023).

[12] Babar, R., et al. Quantum sensor in a single layer van der Waals material. *arXiv 2111.09589* (2021).

[13] Oniuzhuk, M., et al. Probing the coherence of solid-state qubits at avoided crossings, *PRX Quantum*, **2**, 010311 (2021).

[14] Bayliss, S. L., et al. Enhancing Spin Coherence in Optically Addressable Molecular Qubits through Host-Matrix Control, *Phys. Rev. X.,* **12**, 031028 (2022).

[15] Mendelson, N., et al. Identifying carbon as the source of visible single-photon emission from hexagonal boron nitride. *Nat. Mater.*, **20**, 321 (2021).

[16] Benedek, Z., Babar, R., Ganyecz, Á. *et al.* Symmetric carbon tetramers forming spin qubits in hexagonal boron nitride. *npj Comput. Mater.,* **9**, 187 (2023).

[17] Gottscholl, A., et al. Initialization and read-out of intrinsic spin defects in a van der Waals crystal at room temperature. *Nat. Mater.,* **19**, 540 (2020).

[18] Sajid, A., Reimers, J. R., and Ford, M. J. Defect states in hexagonal boron nitride: Assignments of observed properties and prediction of properties relevant to quantum computation. *Phys. Rev. B.,* **97**, 064101 (2018).

[19] Mackoit-Sinkeviciene, M., Maciaszek, M., Van de Walle, C. G. and Alkauskas, A. Carbon dimer defect as a source of the 4.1 eV luminescence in hexagonal boron nitride. *Appl. Phys. Lett*., **115**, 212101 (2019).

[20] Auberger, P. and Gali, A. Towards ab initio identification of paramagnetic substitutional carbon defects in hexagonal boron nitride acting as quantum bits. *Phys. Rev. B*., **104**, 075410 (2021).

[21] Fischer, M. et al. Combining experiments on luminescent centres in hexagonal boron nitride with the polaron model and ab initio methods towards the identification of their microscopic origin. *Nanoscale*, **15**, 14215 (2023).
